# Experimental investigation and development of a deep learning framework to predict process-induced surface roughness in additively manufactured aluminum alloys

**DOI:** 10.1007/s40194-022-01445-8

**Published:** 2022-12-20

**Authors:** Waqas Muhammad, Jidong Kang, Olga Ibragimova, Kaan Inal

**Affiliations:** 1 Department of Mechanical and Mechatronics Engineering, University of Waterloo, Waterloo, N2L 3G1 Canada; 2 CanmetMATERIALS, 183 Longwood Road South, Hamilton, L8P 0A5 Canada

**Keywords:** Artificial intelligence, Deep learning, Additive manufacturing, Laser powder bed fusion, Surface roughness

## Abstract

A deep learning framework is developed to predict the process-induced surface roughness of AlSi10Mg aluminum alloy fabricated using laser powder bed fusion (LPBF). The framework involves the fabrication of round bar AlSi10Mg specimens, surface topography measurement using 3D laser scanning profilometry, extraction, coupling, and streamlining of roughness and LPBF processing data, feature engineering to select the relevant feature set and the development, validation, and evaluation of a deep neural network model. A mix of core and contour-border scanning strategies are employed to fabricate four sets of specimens with different surface roughness conditions. The effects of different scanning strategies, linear energy density (LED), and specimen location on the build plate on the resulting surface roughness are discussed. The inputs to the deep neural network model are the AM process parameters (i.e., laser power, scanning speed, layer thickness, specimen location on the build plate, and the *x*,*y* grid location for surface topography measurements), and the output is the surface profile height measurements. The proposed deep learning framework successfully predicts the surface topography and related surface roughness parameters for all printed specimens. The predicted surface roughness (${S}_{a}$) measurements are well within 5% of experimental error for the majority of the cases. Moreover, the intensity and location of the surface peaks and valleys as well as their shapes are well predicted, as demonstrated by comparing roughness line scan results with corresponding experimental data. The successful implementation of the current framework encourages further applications of such machine learning-based methods toward AM material development and process optimization.

## Introduction

Aluminum alloys are the lightest structural engineering materials and are typically used in aerospace and automotive applications because of their excellent specific strength, corrosion resistance, electrical and thermal conductivity, recyclability, and esthetic appearance [1]. Technological advancements in alloy design and processing over the past few decades have led to the development of a wide range of aluminum alloys suitable for different engineering applications. Traditionally, aluminum components have been produced by conventional casting, forging, hot-rolling, or extrusion methods. In general, these processes tend to produce components with coarse-grain microstructures, which adversely affects their mechanical properties [2]. Furthermore, the costs associated with tooling and production lead times are prohibitive.

In these regards, additive manufacturing (AM) provides an alternative novel manufacturing route that enables the fabrication of near-net-shape parts without intricate tooling requirements and minimal raw material utilization [3]. Among all additive techniques, selective laser melting (SLM) has shown ample fabrication potential [4]. The process employs a high-power laser to selectively melt the powder and create parts layer-by-layer. Unlike conventional manufacturing approaches, the SLM process offers significant time and cost savings through rapid prototyping and shortening of production lead times [5]. Furthermore, due to the high cooling rates associated with the SLM process, the microstructural features (such as grain size) are generally much finer, promoting superior mechanical behavior [6]. However, one of the major issues with SLM-produced materials is the process-induced surface roughness that can have a detrimental impact on the overall surface quality and the tribological behavior of as-built components [7]. The in-service performance (such as fatigue life) of these materials is affected by the surface condition which in effect is controlled by the selection of laser processing parameters [8, 9]. Many factors such as laser power, layer thickness, print orientation, scanning speed, hatch spacing, and powder size affect surface roughness. The relationship between process parameters and the resulting surface roughness of these alloys is a topic of significant interest and needs further research.

Numerous studies have been performed to examine the effects of process parameters on the surface roughness of aluminum alloys, specifically AlSi10Mg alloy, produced by SLM [10–12]. In particular, Calignano et al. [12] conducted a study on the effects of process parameters such as laser power, hatch spacing, and scanning speed on the surface roughness of AlSi10Mg parts and observed that laser scanning speed has the highest impact on the surface quality. Mohammadi and Asgari [10] studied the effects of laser parameters on the surface roughness of cubic AlSi10Mg specimens by employing different process parameters for upskin and core regions. The authors showed that better surface roughness for horizontal surfaces could be obtained by using high specific energy at the lowest experimental beam offset. Yang et al. [13] studied the effects of laser parameters on the vertical surface roughness of AlSi10Mg components produced by SLM. Their results showed that an appropriate linear energy density (LED) could be used to decrease the vertical surface roughness by more than 70%. More recently, Yang et al. [14] investigated the impact of process parameters on the overhanging surface roughness of AlSi10Mg specimens by using different scanning parameters for contour and infill regions. It is concluded that the effects of each process parameter on overhanging surface roughness are not pre-set but in fact change with variations in the build angle. The work further suggests that the contour scanning speed is the dominant factor influencing the overhanging surface roughness. Other researchers have employed laser surface re-melting (LSR) techniques, where the same slice is scanned multiple times before recoating the next powder layer to improve the surface quality of printed components at the expense of added printing time and cost [7, 15–17]. However, the effects of re-melting process parameters on the surface roughness, microstructure, and mechanical properties are not well understood and are an area of active research [18].

Considering the existing research, the process optimization of SLM aluminum alloys is a complex operation due to the high thermal conductivity and reflectivity of the material [12]. Furthermore, the induced surface roughness is controlled by the synergistic effect of numerous process parameters that vary considerably depending on their applied levels. Therefore, understanding the interactions between process parameters and the resulting surface roughness is of great significance for SLM process optimization. In these respects, machine learning (ML)-based approaches provide an effective medium to explore, understand, and establish connections between process parameters and the resulting process-related attributes such as surface roughness. Application of such approaches is becoming more common in the area of computational materials science, with an aim to identify essential material or process parameters and model complex material relationships.

There have been numerous successful applications of machine learning approaches in the area of computational mechanics and materials science. More specifically, ML approaches such as artificial neural network (ANN) have been used to model the constitutive behavior of engineering metals at different strain rates and temperatures [19] to model aspects of plastic deformation and localization [20], to predict the forming limit diagrams [21], to model the multi-axial plasticity behavior [22], and to model the fatigue behavior of engineering materials [23]. More recently, Ibragimova et al. [24] employed an ensemble of ANNs to predict the non-monotonic behavior and texture evolution of face-centered cubic (FCC) polycrystalline materials. Machine learning approaches such as feed forward neural network (FFNN), convolutional neural network (CNN), deep belief network (DBN), *k*-means clustering, support vector machine (SVM), and random forest (RF) have been applied in additive manufacturing to design new materials [25–27], to optimize topology [28, 29], to predict porosity [30–32], to monitor printing process for quality assurance [33–35], to predict thermal history during printing [36, 37], to construct process maps [38, 39], to predict melt pool dimensions [40], to classify melting states [41], and to detect process-induced defects [42–44]. Recently, Muhammad et al. [45] proposed a machine learning framework to model the evolution of local strains, plastic anisotropy, and fracture in AlSi10Mg alloy produced by SLM. Even though prediction and control of the surface roughness of AM parts is of extreme importance, limited effort has been given to develop machine learning-based frameworks to link process parameters to the induced surface roughness.

In the present work, firstly, an experimental study is performed to investigate the connections between laser process parameters and the process-induced surface roughness of AlSi10Mg alloy fabricated using laser powder bed fusion (LPBF) technology. For this purpose, different process parameters combined with a mix of core and contour printing strategies are employed to print cylindrical specimens with different as-fabricated roughness conditions. An emphasis is placed on investigating the relationship between laser parameters such as power, scanning speed, point distance, exposure time, print location, and the resulting surface roughness. Next, a deep learning framework is developed to model and predict the process-induced surface roughness of AlSi10Mg alloy. A successful application of the framework would assist in understanding the connections between process parameters and the resulting surface roughness and would further promote the application of machine learning methods to model microstructure–property–performance relationships for additively manufactured alloys.

## Experimental procedures

### Material and 3D printing

**Fig. 1:**
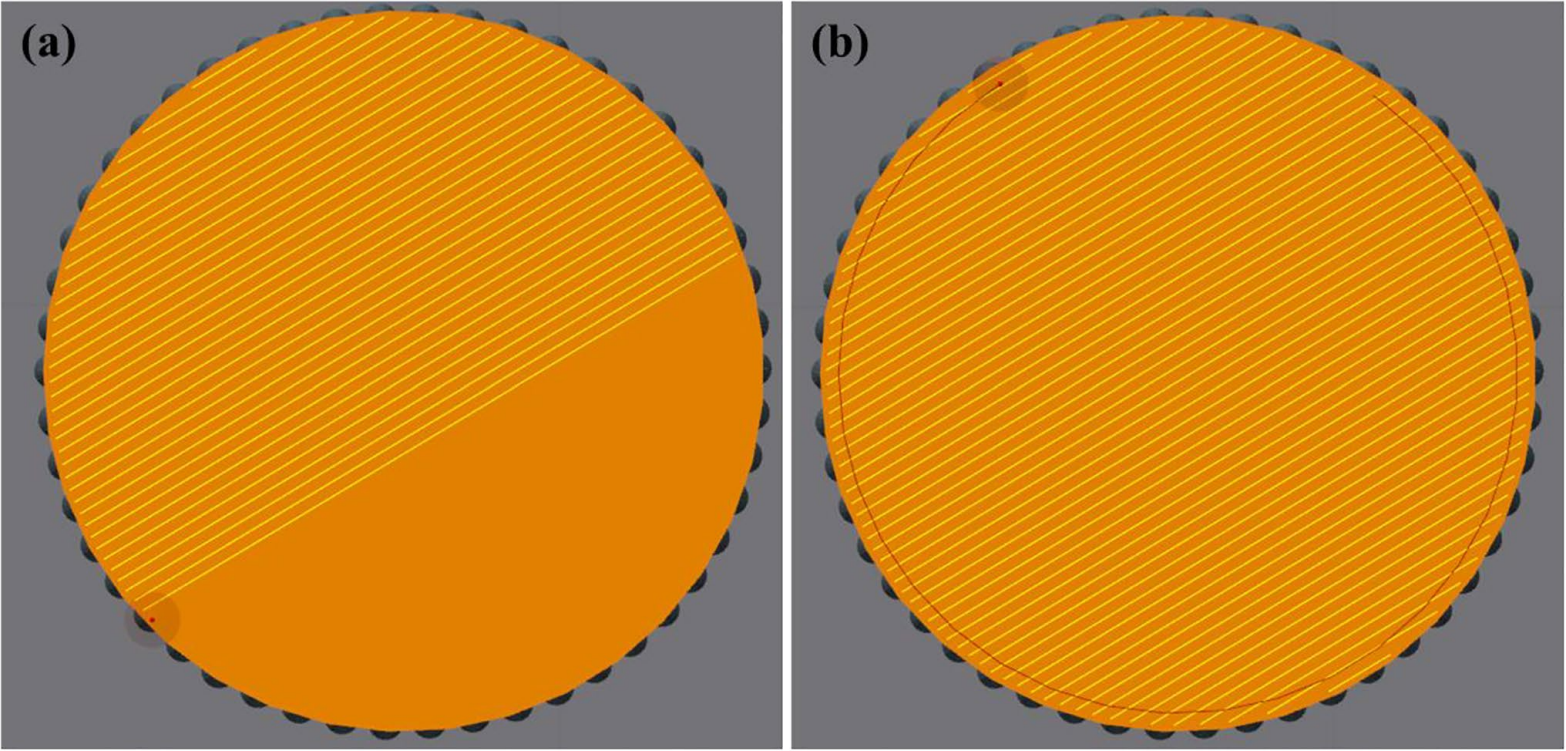
Laser scanning strategy for (**a**) the entire cross-section core region and (**b**) the contour-border region

A pre-alloyed AlSi10Mg aluminum alloy powder with a mean particle size of approx. 40 μm was used in the current work. The chemical composition of the AlSi10Mg powder is given in Table 1. A high weight percentage of silicon helps in improving the strength and hardness of the alloy through the precipitation of Mg_2_Si particles [46]. A total of 40 round bar specimens (i.e., 4 sets of 10 specimens each) were fabricated using the Renishaw AM400 LPBF machine. The printing was performed within a controlled inert Argon atmosphere having an oxygen content of less than 0.1%. A mix of core and contour-border scanning strategies were employed to produce specimens with similar core density and varying surface roughness conditions. As shown in Fig. 1a, the entire cross-sectional or core region of the specimen was first scanned using the core scanning parameters. Next, the contour-border region of the cross-section was scanned once more in a circular manner prior to feeding the next powder layer. This in-situ re-melting close to the border region was performed to improve the surface roughness of as-fabricated specimens. The core recipe (i.e., LPBF process parameters) for the first three sets of specimens were identical, whereas a different contour-border recipe was used for each. The fourth set of specimens was printed with a different core recipe, whereas the contour-border recipe used was the same as that used for specimens in set 2. A different core recipe was chosen to produce specimens with different core densities. The core and contour process parameters for the different sets of specimens are given in Tables 2 and 3, respectively.

**Table 1:** Chemical composition (max. wt%) of AlSi10Mg powder

Si	Mg	Fe	Mn	Cu	Ni	Ti	Zn	Al
9–11	0.25–0.45	0.25	0.1	0.05	0.05	0.15	0.1	Bal

**Table 2:** LPBF process parameters for the core scan of different sets of specimens

Specimen set no	Laser power (W)	Point distance (μm)	Exposure time (μs)	Scanning speed (mm/s)	Hatching distance (μm)	Hatch rotation angle (˚)	Layer thickness (μm)
1–3	176.8	60	110	500	142	67	30
4	255.3	60	50	1000	142	67	30

**Table 3:** LPBF process parameters for the contour-border scan of the different sets of specimens

Specimen set no	Laser power (W)	Point distance (μm)	Exposure time (μs)	Scanning speed (mm/s)	Contour distance from hatch border (μm)	Hatch rotation angle (˚)	Layer thickness (μm)
1	325.5	60	70	750	142	67	30
2	270	60	50	1000	142	67	30
3	212	60	110	500	142	67	30
4	270	60	50	1000	142	67	30

**Fig. 2:**
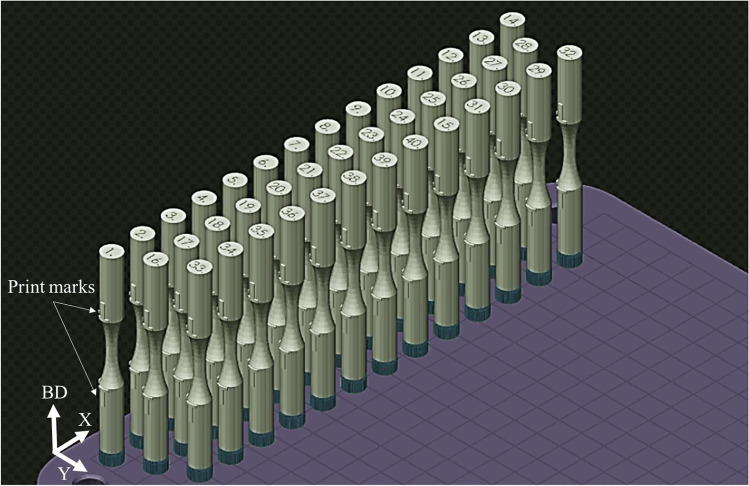
Location of the 40 round bar specimens on the base plate. Note that each specimen is marked with specimen no. between 1 and 40

**Fig. 3:**
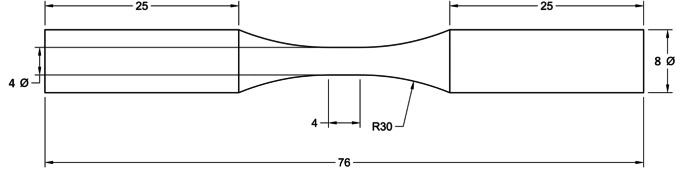
Geometry of the round bar specimen. All dimensions in mm

Figure 2 shows the location of the 40 round bar specimens on the base plate. All specimens were printed with their loading axis aligned with the build direction (Z). Each specimen was given a distinct specimen no. between 1 and 40. Specimens from different sets are randomly distributed on the base plate to minimize experimental bias. The specimen numbering corresponding to each set is given in Table 4, and the geometry of the printed round bar specimen is shown in Fig. 3. Some of the specimens were not printed correctly due to a recoater issue, and they are excluded from the analysis.

**Table 4:** Specimen no. corresponding to each of the four printed sets

Set no	Specimen no
1	2, 6, 10, 14, 22, 26, 30, 38
2	1, 5, 9, 13, 17, 21, 25, 29, 37
3	3, 7, 11, 15, 19, 23, 27, 31, 39
4	4, 8, 12, 16, 20, 24, 28, 32, 40

### Surface roughness measurements

3D laser confocal microscope Keyence VK-X250 was used to measure the surface roughness of as-fabricated specimens. The imaging process takes surface height measurements using the point illumination method, where a laser beam is scanned across the sample in a raster pattern at incremental vertical displacements. The vertical resolution is dependent on the aperture of the objective lens. The measured height data was then analyzed using Keyence Multi-file Analyzer software. Image processing procedures were applied to account for the curved surface of the cylindrical samples, and surface roughness parameters are calculated. The surface roughness measurements were performed using an objective lens of 20 × magnification with a z-pitch of 0.20 µm. Approximately 4.7 mm × 1.4 mm of surface area was scanned within the central gage section of each specimen. The nominal scan step size was set to 1.4 µm which corresponds to 3345 × 1024 pixels within the surface map. Efforts were made to scan approximately similar regions across each specimen using print marks on specimens, as shown in Fig. 2. Each specimen was aligned under the confocal microscope using print marks as guidance prior to scanning the central gage section.

### Experimental results and discussion

**Fig. 4:**
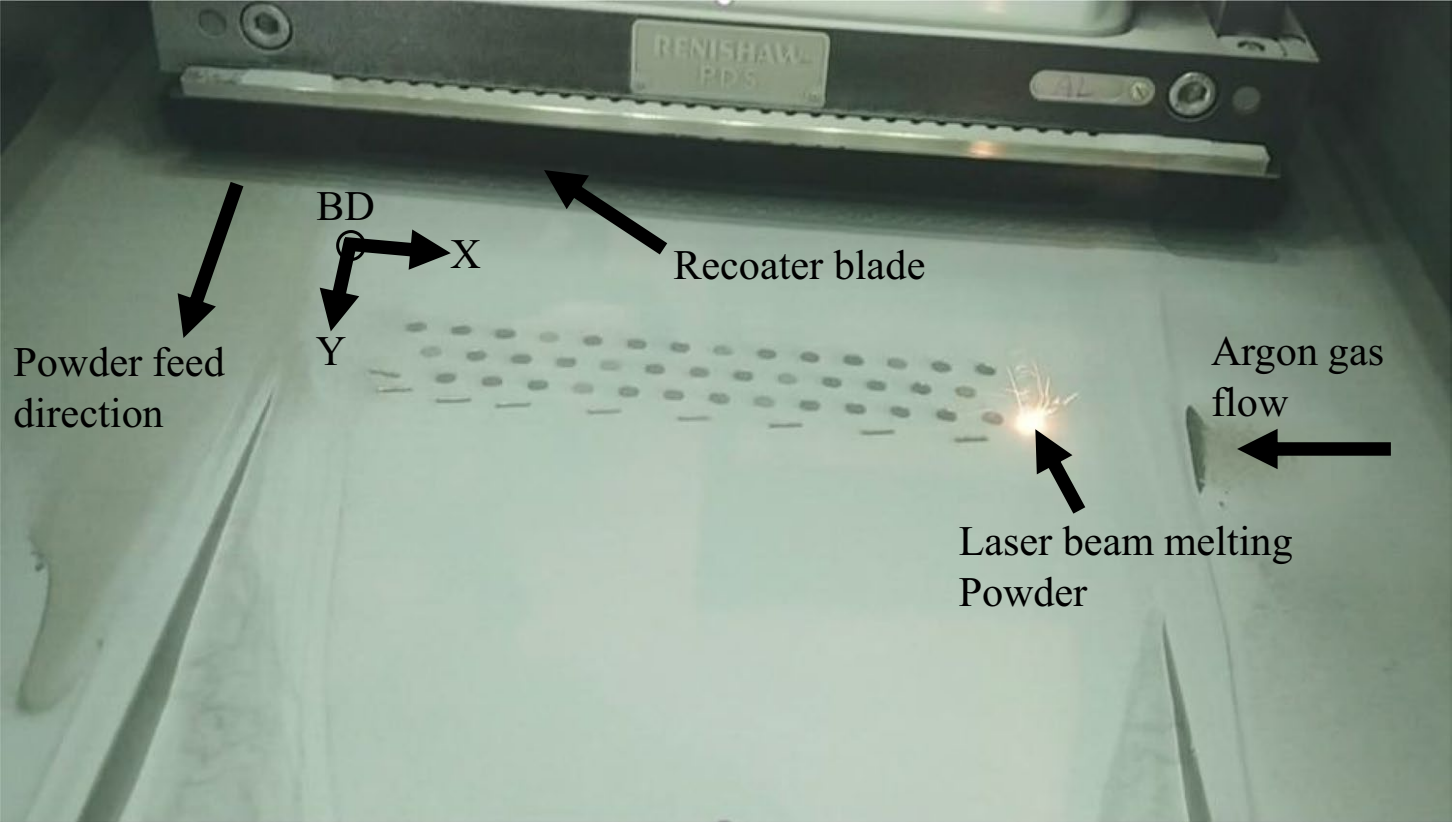
LPBF Printing of AlSi10Mg round bar specimens

**Fig. 5:**
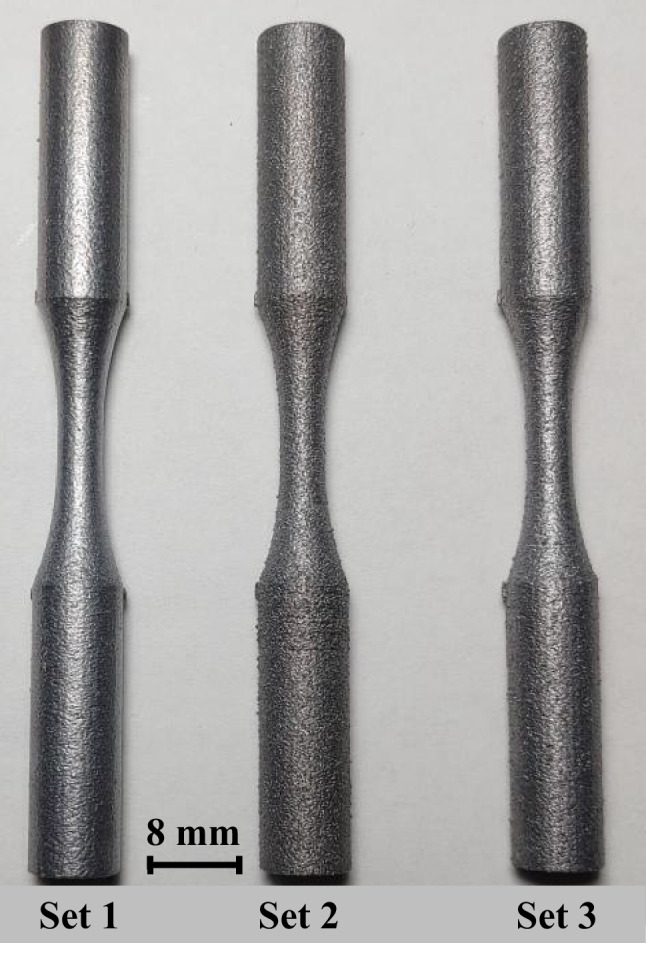
AlSi10Mg round bar specimens

Figure 4 shows a snapshot image during the LPBF printing of AlSi10Mg round bar specimens. The powder feed direction, inert gas flow direction, and position of the specimens on the build plate are shown. A single as-fabricated specimen from each of the first three printed sets is shown in Fig. 5. The specimens in set 4 had a similar physical appearance to those in set 3 and are not shown in Fig. 5. It is visually apparent that set 1 specimens have the lowest surface roughness among the three sets of specimens.

**Fig. 6:**
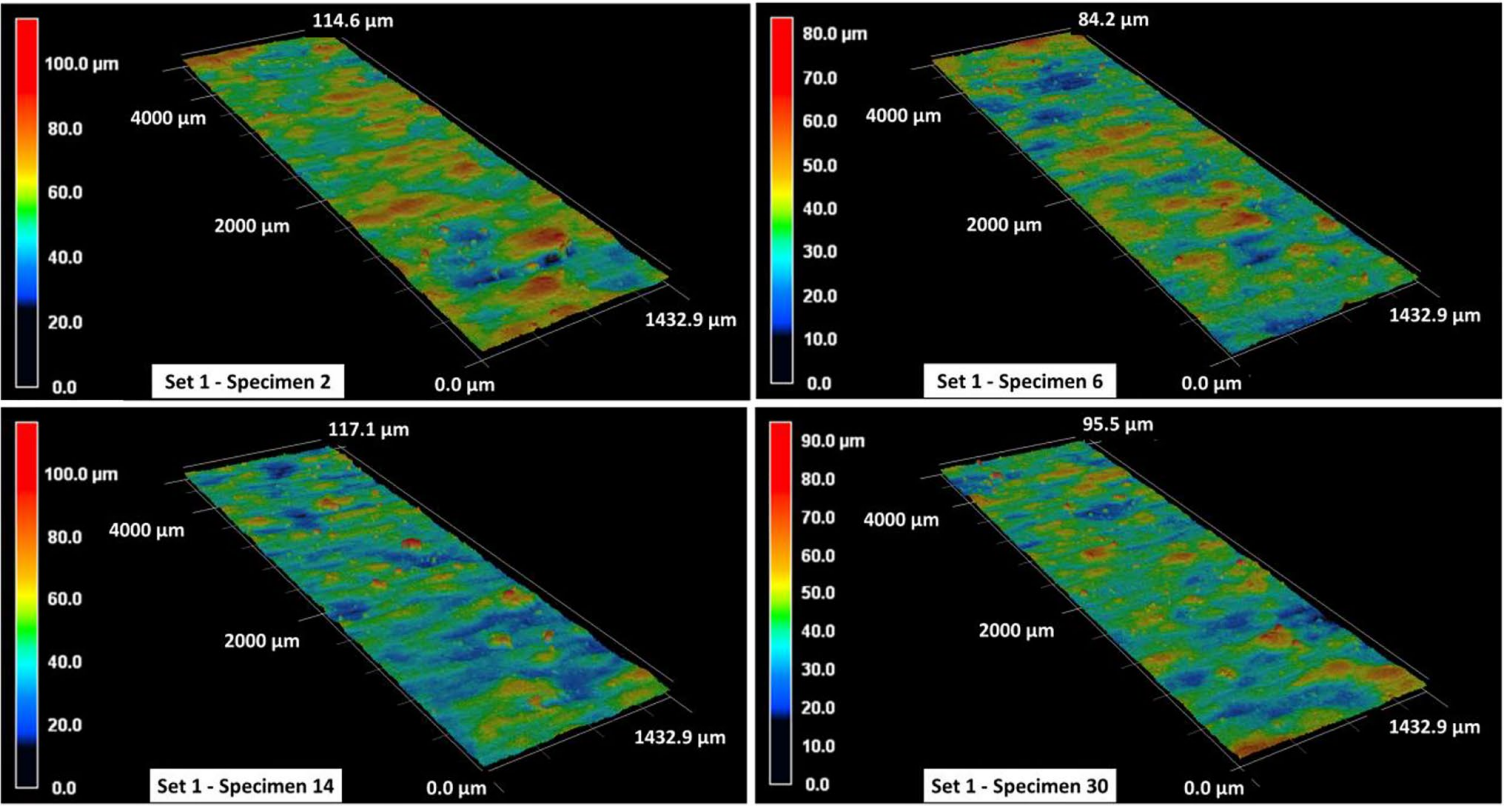
Surface roughness profile for set 1 specimens

**Fig. 7:**
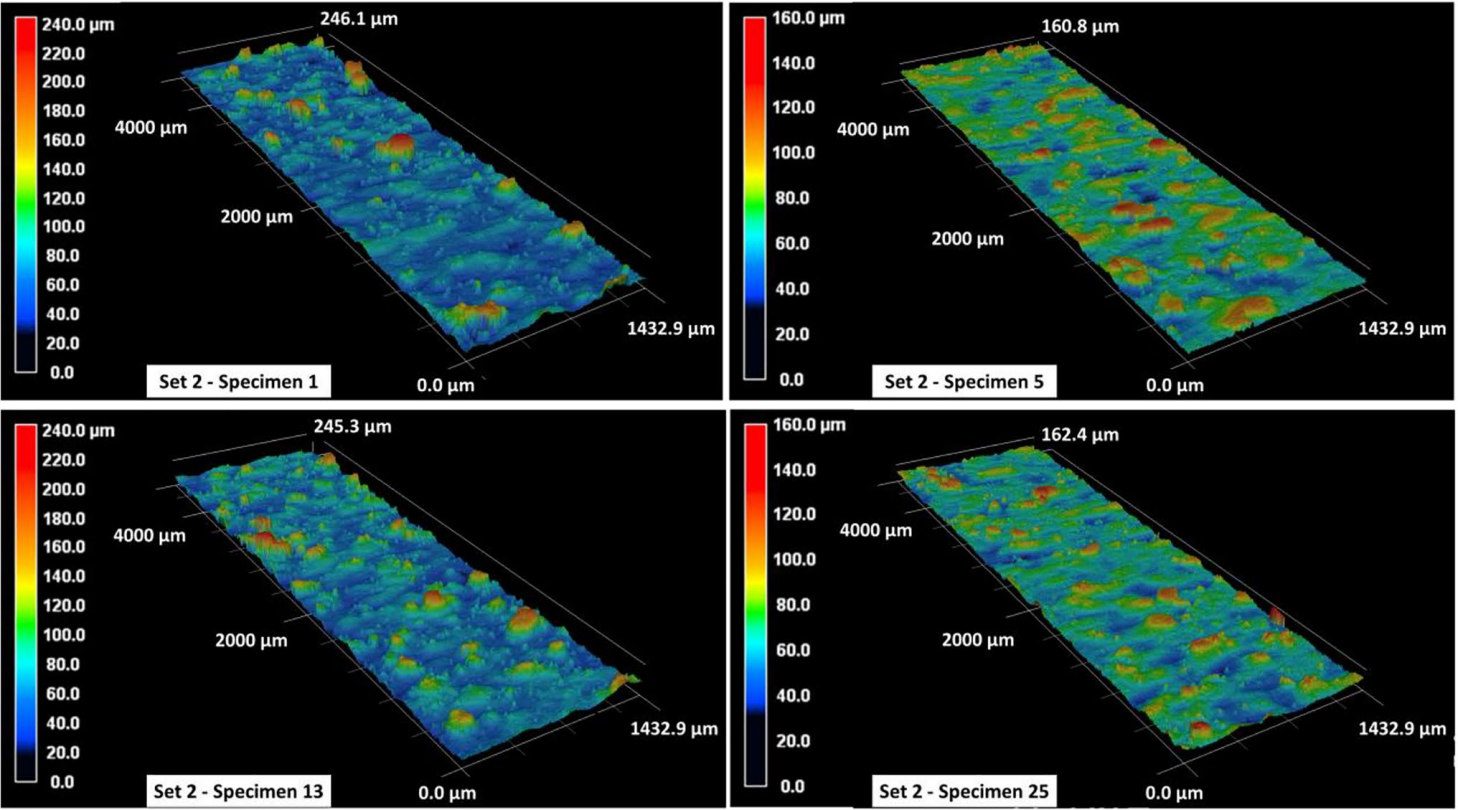
Surface roughness profile for set 2 specimens

**Fig. 8:**
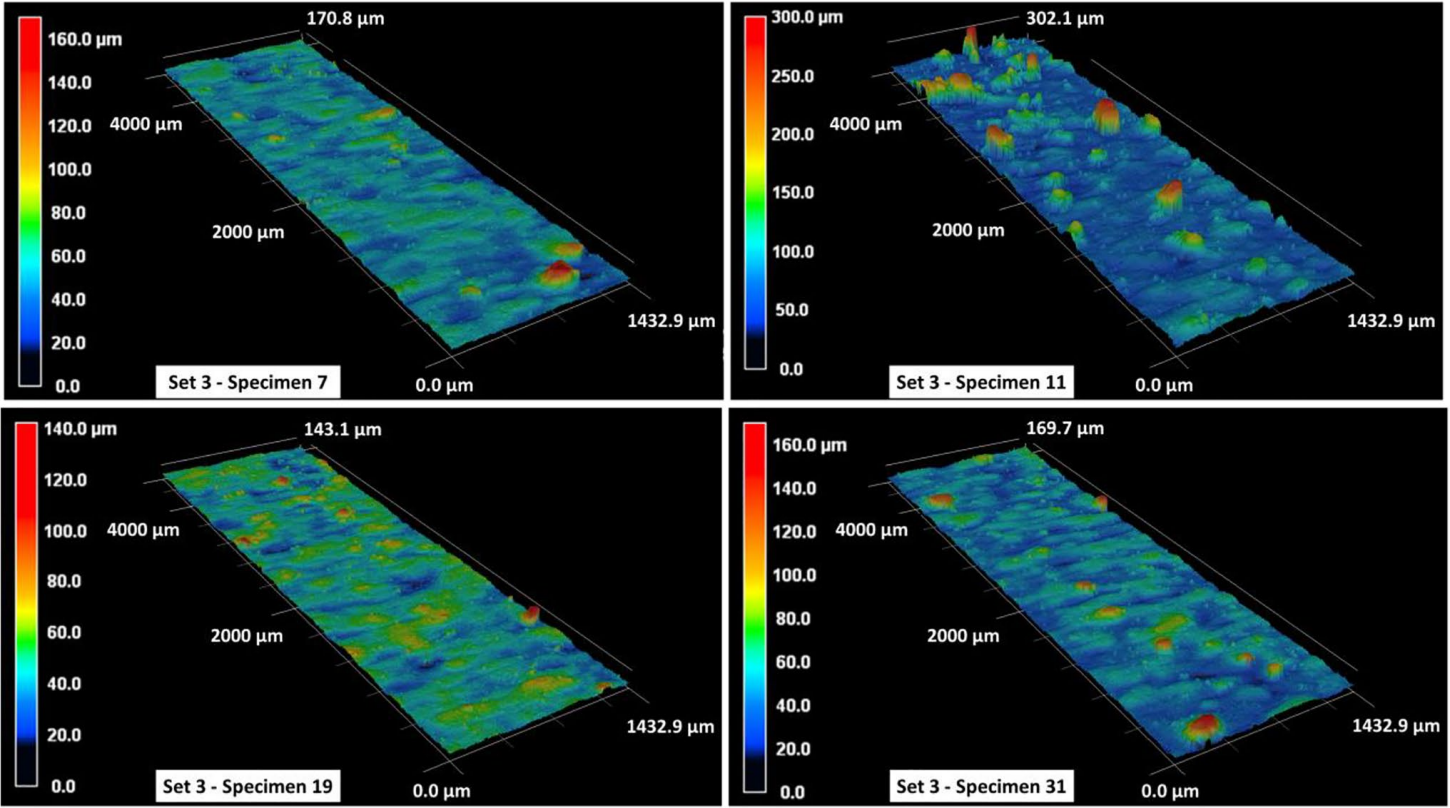
Surface roughness profile for set 3 specimens

The surface roughness contour maps and the corresponding roughness parameters for specimens in set 1 are given in Fig. 6 and Table 5, respectively. In Table 5, the arithmetical mean height ${S}_{a}$, the root mean square height ${S}_{q}$, the maximum peak height ${S}_{p}$, the maximum valley depth ${S}_{v}$, and the maximum height ${S}_{z}$ within a definition area $A$ are calculated as follows:

1 $${S}_{a}=\frac{1}{A} \iint \left|z\left(x,y\right)\right|dxdy$$

2 $${S}_{q}=\sqrt{\frac{1}{A} \iint {z}^{2}\left(x,y\right)dxdy}$$

3 $${S}_{p}=\mathrm{max\;} z\left(x,y\right)$$

4 $${S}_{v}=\left|\mathrm{min\;} z\left(x,y\right)\right|$$

5 $${S}_{z}= {S}_{p}+ {S}_{v}$$

where $z\left(x,y\right)$ represents the measured height value corresponding to a given $x,y$ grid point within the definition surface area $A$. The surface roughness behavior for specimens in set 1 is fairly consistent with an approximate ${S}_{a}$ value of 6.5 to 7 μm. The average maximum peak height is in the vicinity of 60 μm, whereas the average maximum valley depth across all specimens is approx. 40 μm. The roughness profiles in Fig. 6 also exhibit some variations in surface profile heights with maximum height measurements ranging between 85 and 100 μm. These variations in height measurements are more significant, ranging between 160 and 240 μm, for specimens in set 2, as shown in Fig. 7. Furthermore, the average surface roughness for all specimens in set 2 is also comparatively higher with an ${S}_{a}$ value that ranges between 10 and 12 μm (see Table 6). There are few exceptions (such as specimen 13 with an ${S}_{a}$ of 18.71 μm), and those will be discussed later. Excluding the exceptions, the surface roughness ${S}_{a}$ for specimens in set 3 ranges between 8 and 10 μm and that for specimens in set 4 ranges between 9 and 12 μm (see Tables 7 and 8). It is also noted that the maximum peak height ${S}_{p}$ and maximum valley depth ${S}_{v}$ values are higher for specimens in set 3 and set 4 as compared to those in set 1. This is also apparent from the surface profile map of specimen 11 in Fig. 8, where one can see the presence of several aggressive peaks and valleys.

**Table 5:** Surface roughness measurements for specimens in print set no. 1

$\mathrm{Set no}.$	$\mathrm{Specimen no}.$	${S}_{a} \left(\mu m\right)$	${S}_{q} \left(\mu m\right)$	${S}_{p} \left(\mu m\right)$	${S}_{v} \left(\mu m\right)$	${S}_{z} \left(\mu m\right)$
1	2	7.19	9.20	57.20	57.11	114.30
1	6	6.65	8.50	48.91	35.35	84.25
1	10	6.72	8.65	59.57	40.32	99.89
1	14	8.37	10.76	73.96	43.36	117.32
1	22	6.37	8.21	65.46	38.98	104.44
1	26	7.01	9.46	69.87	41.93	111.80
1	30	6.50	8.46	53.88	41.55	95.43
1	38	6.58	8.47	60.36	35.32	95.69

**Table 6:** Surface roughness measurements for specimens in print set no. 2

$\mathrm{Set no}.$	$\mathrm{Specimen no}.$	${S}_{a} \left(\mu m\right)$	${S}_{q} \left(\mu m\right)$	${S}_{p} \left(\mu m\right)$	${S}_{v} \left(\mu m\right)$	${S}_{z} \left(\mu m\right)$
2	1	16.57	25.17	172.40	73.53	245.93
2	5	11.03	14.35	88.84	72.12	160.96
2	9	11.64	16.28	141.82	51.68	193.50
2	13	18.71	26.14	173.23	72.00	245.23
2	17	11.77	16.20	141.93	72.49	214.42
2	21	11.05	15.92	153.10	50.22	203.33
2	25	11.26	15.07	91.45	70.95	162.40
2	29	12.91	18.41	143.00	56.30	199.30
2	37	9.96	12.77	87.90	56.47	144.37

**Table 7:** Surface roughness measurements for specimens in print set no. 3

$\mathrm{Set no}.$	$\mathrm{Specimen no}.$	${S}_{a} \left(\mu m\right)$	${S}_{q} \left(\mu m\right)$	${S}_{p} \left(\mu m\right)$	${S}_{v} \left(\mu m\right)$	${S}_{z} \left(\mu m\right)$
3	3	15.08	23.29	183.88	80.18	264.05
3	7	9.34	13.14	117.91	52.84	170.74
3	11	21.63	33.49	222.35	79.94	302.29
3	15	9.20	12.98	112.65	55.29	167.94
3	19	8.30	10.85	95.68	47.77	143.46
3	23	9.70	15.48	185.94	56.37	242.31
3	27	19.36	28.03	159.65	63.52	223.17
3	31	9.98	14.13	113.34	56.33	169.67
3	39	8.37	14.74	211.48	48.22	259.71

**Table 8:** Surface roughness measurements for specimens in print set no. 4

$\mathrm{Set no}.$	$\mathrm{Specimen no}.$	${S}_{a} \left(\mu m\right)$	${S}_{q} \left(\mu m\right)$	${S}_{p} \left(\mu m\right)$	${S}_{v} \left(\mu m\right)$	${S}_{z} \left(\mu m\right)$
4	4	12.06	17.77	133.82	54.66	188.48
4	8	11.55	16.96	157.51	69.57	227.08
4	12	14.57	21.29	158.09	66.39	224.48
4	16	14.42	20.72	185.15	63.58	248.73
4	20	9.58	12.97	85.52	50.31	135.83
4	24	10.99	15.52	152.65	47.18	199.84
4	28	20.22	29.70	176.37	64.72	241.09
4	32	18.30	26.63	172.33	65.14	237.48
4	40	10.93	15.84	161.87	54.47	216.34

It is noted that a few specimens within each of the four sets have much higher surface roughness as compared to the remaining specimens within those sets. For instance, specimen 14 from set 1 has an ${S}_{a}$ of 8.37 μm which is higher than all remaining specimens in that set. The difference is more significant for specimens in set 2 with specimen 1 and specimen 13 having an ${S}_{a}$ of 16.57 μm and 18.71 μm, respectively, which is significantly higher than the remaining specimens within that set. Similar observations are made for specimen 3, specimen 11, and specimen 27 from set 3 and specimen 12, specimen 16, specimen 28, and specimen 32 from set 4. Upon careful observation, it is noticed that these specimens were located at the right and leftmost corners of the build plate. It is noted that the direction of gas flow in the chamber (which is from left to right, as shown in Fig. 4) and melt pool dynamics could cause powder particle spattering which could lead to this behavior. The spattering of powder particles is further aided by the lightweight nature of the aluminum powder. This spattering of powder particles or melt pool spattering can be observed in Fig. 4. It is suggested that the spattering and accumulation of powder particles could lead to powder agglomeration, resulting in loss of powder in some areas and redistribution of the powder particles on the print bed, which can alter the processing of successive powder layers and laser tracks [47]. The powder agglomeration and altering of powder redistribution on the powder bed could impact the printing process due to variations in powder layer thickness and laser energy absorption and may ultimately impact the surface roughness of the printed specimens. Thus, even though the specimens within each set are printed using the same laser parameters, their actual location on the build plate can also impact the surface roughness.

Excluding the specimens in the leftmost or rightmost corner of the build plate, it is observed that specimens within set 2 and set 4 have a similar surface roughness (i.e., ${S}_{a}$ ranging between 9 and 12 μm). As mentioned previously, both sets were printed using the same contour border and different core parameters (see Tables 2 and 3). This suggests that changing the core printing parameters does not have any significant impact on the final surface appearance and one can achieve a lower surface roughness by simply altering the contour-border parameters, as is the case for the first three printed sets. Upon observation of surface roughness profiles of several specimens across multiple sets, there is no noticeable correlation between the location of peak and valleys across different specimens. However, it is observed that the maximum peak height ${S}_{p}$ values are always greater than the maximum valley depth ${S}_{v}$ across all specimens (see Tables 5, 6, 7, and 8). The difference between ${S}_{p}$ and ${S}_{v}$ is minimal for specimens in set 1 with the smoothest surface appearance and is rather significant for specimens in set 2, set 3, and set 4. It is suggested that this difference might be related to the partly unfused and agglomerated powder particles stuck to the specimen surface, as typically observed for AM parts with high surface roughness.

The combined effect of laser process parameters on defects formation and surface roughness is typically studied using a so-called factor the volume energy density (VED) which is defined as.

6 $$VED= {~}^{P}\!\left/ \!{~}_{LV{h}_{d}}\right.$$

where $P$ represents the laser power, $L$ is the layer thickness, ${h}_{d}$ is the hatch distance, and $V$ is the beam velocity or scan speed which is a function of laser point distance and exposure time. In the present work, since only two independent parameters (i.e., $P$ and $V$) were varied while printing different sets, a simpler definition of the laser energy, the so-called linear energy density (LED), is considered and is defined as follows [17]:

7 $$LED= {~}^{P}\!\left/ \!{~}_{V}\right.$$

**Fig. 9:**
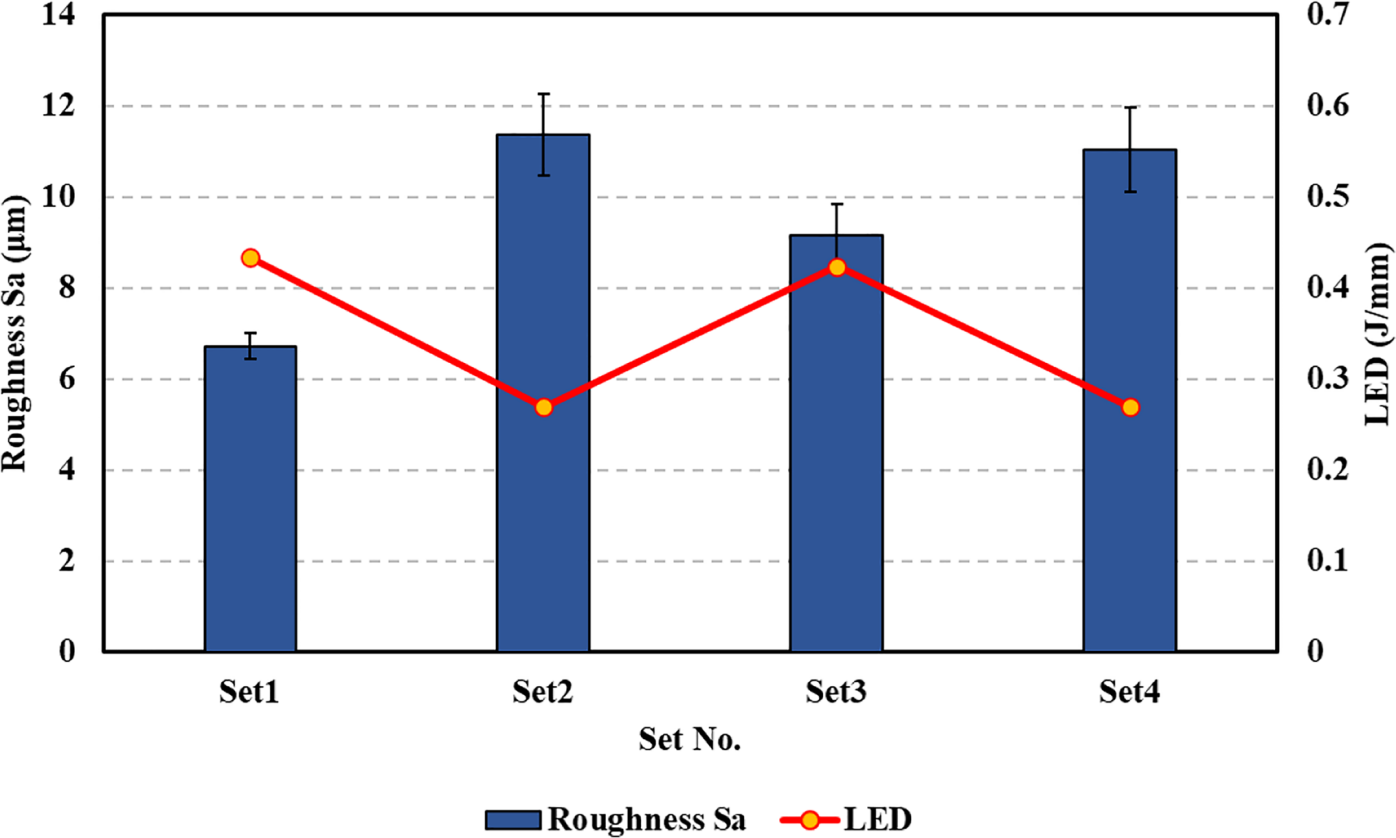
Average surface roughness ${{{S}}}_{{{a}}}$ vs. linear energy density (LED) for the four printed specimen sets

Figure 9 shows a comparison of the surface roughness parameter ${S}_{a}$ and the applied LED for the four printed sets. The average surface roughness value ${S}_{a}$ for each set is reported, and the specimens that are close to the left or rightmost edges of the build plate are excluded from the analysis. The LED values are calculated using the contour-border process parameters. In Fig. 9, specimens in set 2 and set 4 have the same linear energy density of 0.27 J/mm as they were printed using the same contour-border parameters and consequently have a similar average surface roughness (i.e., 11.37 μm for set 2 and 11.04 μm for set 4). Specimens in set 1 have the highest energy density of 0.434 J/mm and the lowest average roughness of approx. 6.72 μm. Specimens in set 3 have an energy density of 0.424 J/mm and an average roughness of approx. 9.15 μm. Based on the results, it is observed that the use of higher energy density is vital for achieving lower surface roughness. This behavior is consistent with the results reported by other researchers, where increasing the surface energy density improves the surface finish [7, 10]. However, it is important to carefully select the LED as a significantly higher LED can promote balling effect and the lack of sufficient laser energy density may lead to defects such as porosity and microcracks, thereby impacting the surface quality of the part. It is also interesting to note that the LED for set 1 and set 3 are fairly close (i.e., 0.434 J/mm for set 1 vs. 0.424 J/mm for set 2). These energy densities were obtained by choosing high laser power and scanning speed combination for set 1 and a relatively low laser power and scan speed combination for set 3. Even though the energy density is fairly close for both sets, the high laser power and scan speed combination used for set 1 produces significantly lower roughness. This highlights the fact that employing a similar LED can lead to different surface conditions since the other chosen scanning parameters might be more sensitive to the choice of laser power and scan speed and may play a significant role in controlling the final roughness.

In brief, four sets of specimens with varying surface roughness were fabricated by altering the core and contour-border parameters. It is observed that the choice of core parameters has no significant impact on the surface roughness. The surface roughness is mainly controlled by the contour-border parameters. It is noted that the use of higher LED leads to lower surface roughness. However, the actual surface roughness is a consequence of the synergistic effects of several different printing parameters and may not be properly represented by simple factors such as the LED. Furthermore, it is observed that apart from the choice of printing parameters, the surface roughness of the printed specimens is also influenced by their location on the build plate. The specimens in the vicinity of the right or leftmost edges of the build plate have comparatively higher roughness compared to other specimens within the same print set. Based on these observations, it is noted that the control and prediction of surface roughness of AlSi10Mg specimens is difficult due to the complexity of AM process. In the next section, the experimental data is used to develop and validate a machine learning framework for predicting the surface roughness of AlSi10Mg specimens fabricated using LPBF.

## Deep learning framework development

**Fig. 10:**
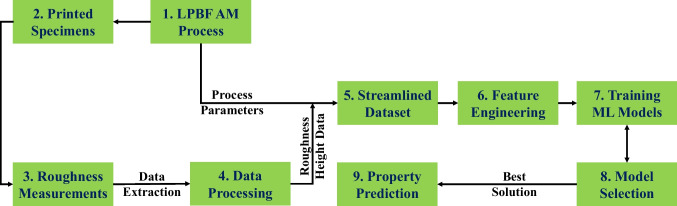
Schematic of the proposed machine learning framework

One of the objectives of the current work is to develop a deep learning framework to model the process-induced surface roughness of AlSi10Mg aluminum alloy fabricated by LPBF. This will be achieved by coupling the methods and processes involved in AM and AI to allow the prediction of local variations in surface roughness that are typical of AM materials. A schematic representation of the overall framework is shown in Fig. 10. It involves 3D printing of AlSi10Mg specimens with different surface roughness conditions using LPBF, measurement of the process-induced surface roughness using 3D laser confocal microscopy, extraction, coupling, and streamlining of input data from 3D printing and roughness measurements, feature engineering to choose the relevant features, ANN network selection, training, and evaluation. Information on the generation of dataset, feature selection, and ANN model development are provided next.

### Dataset generation and feature selection

Dataset generation lies at the core of any machine learning framework implementation. It is important that the generated dataset is representative of the problem at hand. In the present work, the dataset consisting of surface roughness profile height measurements is obtained experimentally using laser profilometry. The dataset from surface roughness maps is extracted as roughness profile height measurement $z\left(x,y\right)$ corresponding to a given *x*,*y* grid location on the surface map. This height measurement data from different specimens is combined with the corresponding laser scanning parameters to create a streamlined dataset for training and validation.

Apart from reliable dataset generation, feature engineering also plays a vital role in the successful implementation of any machine learning framework. Feature selection is done to identify the most relevant set of features that can adequately model a given problem at hand. The current material system that is being modeled is an AM alloy with a complex processing-thermal history leading to different process-induced surface roughness. Based on the experimental results, it is noted that the surface roughness is affected by choice of contour-border laser scanning parameters and the location of the printed specimen on the build plate. Accordingly, the selected features of interest are outlined in Table 9 below.

**Table 9:** The relevant features and their description

Feature	Description
$\mathrm{Laser\;parameters}$	Laser power $P$, scan speed $V$, layer thickness $L$
$\mathrm{Specimen\;location }{(S}_{L})$	Location of the specimen on the build plate
$\mathrm{Grid\;location\;}{(G}_{L})$	Location of the point of interest on surface roughness *x*,*y* grid map or profile
$\mathrm{Roughness\;height\;} (Z(x,y))$	Roughness profile height at a given *x*,*y* location on the roughness grid map

**Fig. 11:**
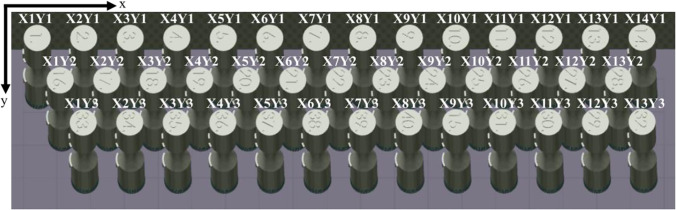
Representation of specimen location on the build plate

In Table 9, the specimen location ${(S}_{L})$ on the build plate is represented by defining a custom *x* and *y* coordinate system, as shown in Fig. 11. Based on its location on the build plate, each specimen is assigned an *X* and *Y* value. This feature is chosen to ensure that the effects of specimen location on the surface roughness can be identified and modeled properly by the machine learning framework.

### ANN model

A subclass of ANN, the deep feedforward neural networks (D-FFNN), also known as multilayer perceptrons (MLPs), are employed in the present study. The goal of a feedforward network is to approximate a continuous function $f$ that maps a given input $x$ to a known output $y$ such that $y= f(x)$. The network architecture consists of an input layer, followed by a single or multiple hidden layers and an output layer. These models are called feedforward since the information flow within the network is in the forward direction, starting with the function being evaluated from $x$ (i.e., input layer), through the intermediate calculations used to define $f$ (i.e., hidden layers), and finally to the output $y$ (i.e., the output layer). Depending on the complexity of the input dataset and linkages between the inputs and outputs, the network may consist of any number of hidden layers with several neurons per layer. The output ${o}_{i}^{l}$ of each neuron is calculated as

8 $${o}_{i}^{l}= \varphi \left(\sum_{j=1}^{q}{\omega }_{ij}^{l}{o}_{j}^{l-1}+{b}_{i}^{l}\right)$$

where the weights ${w}_{ij}^{l}$ of the ${l}^{th}$ layer are multiplied with the output ${o}_{j}^{l-1}$ of the previous $l-1$ layer and corrected for the bias ${b}_{i}^{l}$. In Eq. (8), $\varphi$ represents the activation function and is used to introduce non-linearity into the output of a given neuron to model the complex linkages between the inputs and the outputs. Some of the typical activation functions are the rectified linear unit (ReLU), sigmoid, and hyperbolic tangent functions and are given in Eqs. (9)–(11), respectively.

9 $$\varphi \left(x\right)= \left\{\begin{array}{c}0\;for\;x<0\\ x\;for\;x \ge 0\end{array}\right.$$

10 $$\varphi \left(x\right)= \frac{1}{1+{e}^{-x}}$$

11 $$\varphi (x)= \frac{{e}^{x}- {e}^{-x}}{{e}^{x}+ {e}^{-x}}$$

ReLU in Eq. (9) is the simplest activation function that returns 0 upon receiving a negative input or returns back the input value for any positive value $x$. The output of the sigmoid function (in Eq. (10)) ranges between [0,1] and that of the hyperbolic tangent function (in Eq. (11)) ranges between [− 1,1]. It is noted that the derivatives of the hyperbolic tangent function are larger than those of the sigmoid function, allowing for a faster convergence rate and minimization of the cost function [48].

The function $f$ is approximated by minimizing a cost function $C$ that defines the performance of the so-called training process. The cost function $C$ is typically computed by averaging all the individual losses ${L}_{i}$ across all training samples. The cost function used in the present work is the well-known mean squared error (MSE) function, which is defined as follows:

12 $$C=\frac{1}{n}\sum_{i=1}^{n}{L}_{i} \mathrm{\;where\;} {L}_{i}= \frac{{\Vert {y}_{i}- {o}_{i}\Vert }^{2}}{2}$$

where ${y}_{i}$ is the actual value, ${o}_{i}$ is the predicted value, and $n$ is the total number of training samples. In essence, the optimization of the ANN network is equivalent to minimizing the cost function $C(w, b)$ that in itself is a function of the different network weights and biases. The cost function is minimized using a stochastic gradient descent (SGD) scheme that employs a backpropagation algorithm to iteratively adjust the weights and biases until a minimum of the cost function is found. The partial derivatives of the cost function $C$ with respect to the network weights ${\omega }_{ij}$ are given by

13 $$\frac{\partial C}{\partial {\omega }_{ij}^{l}}=\frac{\partial C}{\partial {o}_{i}^{l}} \frac{\partial {o}_{i}^{l}}{\partial {\omega }_{ij}^{l}}$$

and the new weights are calculated using

14 $${\left({w}_{ij}^{l}\right)}_{\mathrm{new}}={\left({w}_{ij}^{l}\right)}_{\mathrm{old}}-\alpha \frac{\partial C}{\partial {w}_{ij}^{l}}$$

where $\alpha$ is the learning rate that controls the step size of gradient descent. The learning rate must be chosen carefully as a too-small value for the learning rate would lead to longer optimization times and a too-large value would cause convergence issues. In brief, at the beginning of the optimization process, all the network weights and biases are initialized, the inputs are passed through the network, and a preliminary output is determined. Next, the cost function is calculated and minimized iteratively in several training epochs by adjusting the weight and biases using the backpropagation algorithm until the cost function is minimized.

In the current work, the inputs to the ANN model are the laser processing parameters of the contour-border scan such as the laser power $P$, scan speed $V$, layer thickness $L$, specimen location on the build plate, and the *x*,*y* grid location ${(G}_{L})$ of the region of interest for surface roughness prediction. The output of the network is the surface profile height $Z(x,y)$ at a given *x*,*y* grid location ${(G}_{L})$ on the surface. The predicted height variations $Z(x,y)$ are then processed (using Eqs. (1) to (5)) to calculate the required surface roughness parameters. The training process is carried out in python using Keras with TensorFlow backend. The experimental roughness height measurement data from all the specimens is pooled together and shuffled to create an “overall dataset.” This overall dataset is then randomly split (to minimize human bias) into 70% training and 30% test datasets. The training dataset is further split, and approximately 15% of the training dataset (which is 10.5% of the overall dataset) is used for validation purposes to check the accuracy of the model parameters at the end of each training epoch. So, the network is being trained on approx. 59.5% of the overall dataset and the remaining 10.5% is being used for validation purposes only. After the split, there are approximately 71,224,832 and 12,569,088 data samples in the training and validation datasets. Due to the large number of training samples, the training data is fed to the neural network in batches with a batch size of 16,384 samples per batch. Prior to training, the dataset is normalized to ensure that all values lie in a comparable range and to improve the convergence of the gradient descents during optimization. At the beginning of the training process, the glorot uniform kernel is used to initialize all network weights to random values and the optimization process conducted using Adam adaptive learning rate algorithm as it is specifically designed for training deep neural networks [49].

## Analyses and discussion

**Fig. 12:**
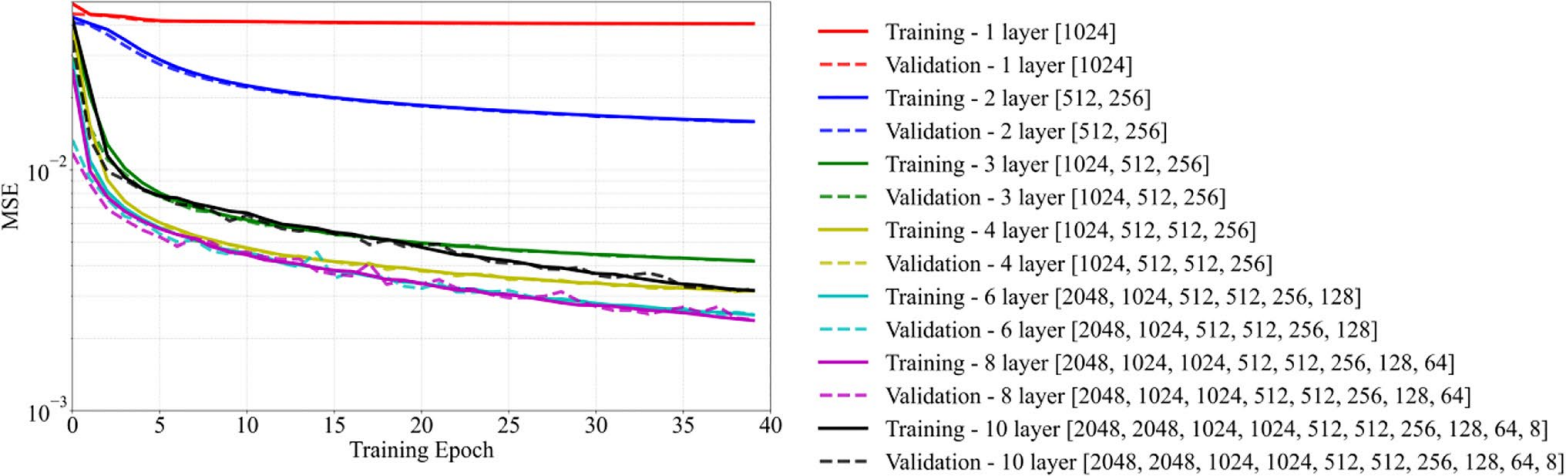
Learning curves for various network architectures used for training. The legend shows the different network architectures (i.e., number of layers – number of neurons per hidden layer inside square brackets)

**Fig. 13:**
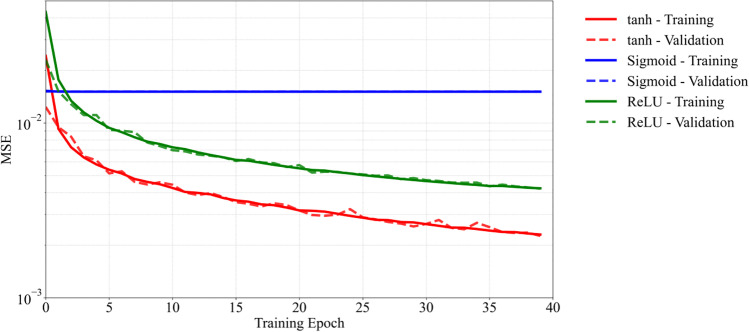
Learning curves for training and validation using typical activation functions

As part of the ANN architecture selection, a grid search methodology is used to train various networks. Several networks are trained with varying numbers of hidden layers, number of neurons per layer, and the choice of activation function and their performances are assessed. Figure 12 shows the network learning curves, presenting the evolution of mean squared error (MSE) as a function of training epochs for the different network architectures. For each training case, the number of hidden layers and the number of neurons per layer are given within the legend in Fig. 12. It is shown that for less than 3 hidden layers, the model is not deep enough to learn on the training dataset and is underfitting. A significant decrease in MSE is observed once the number of hidden layers is increased from 1 to 3. Specifically, the error decreases by order of magnitude from 4 × 10^−2^ to 4 × 10^−3^. The MSE decreases further as the number of hidden layers is increased from 3 to 6 at the expense of increased network complexity. Furthermore, there is no noticeable change in the MSE for networks with hidden layers between 6 and 8. As the number of hidden layers is increased to 10, the network architecture becomes over-complicated and leads to an increase in the training and validation error. Apart from the number of hidden layers, the number of neurons within each subsequent hidden layer is gradually decreased to reduce the complexity of the overall network. Fig. 13 shows the evolution of MSE for training using different activation functions. The network with 6 hidden layers is chosen and re-trained using the hyperbolic tangent (tanh), sigmoid, and ReLU activation functions. The sigmoid activation function performed the worst with the highest MSE that did not decrease any further after a couple of initial training epochs. In contrast, the network with the tanh activation function has the lowest MSE. The results suggest that the ANN architecture with 6 hidden layers along with the tanh activation function is suitable for training the current network.

**Fig. 14:**
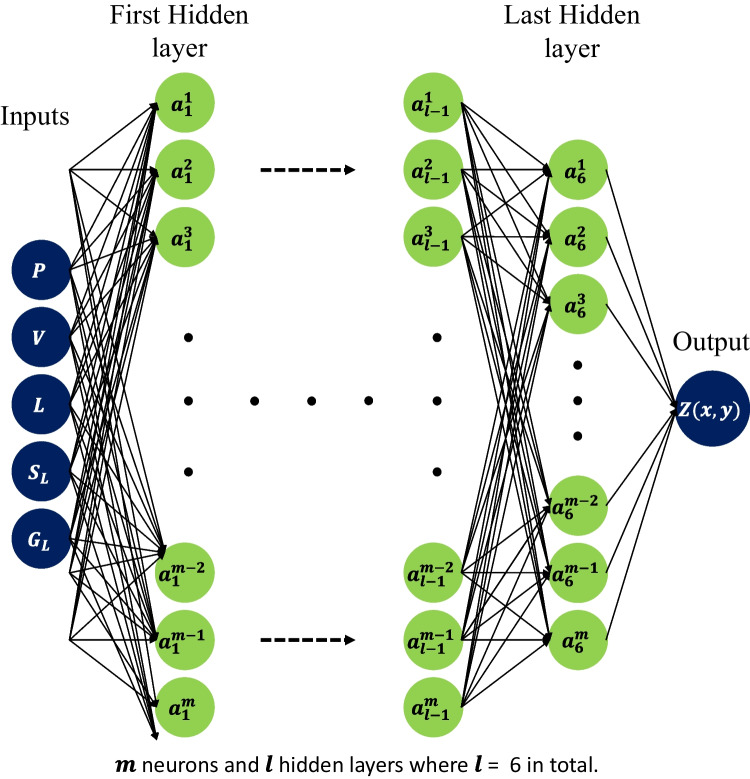
Network architecture of the proposed ANN model

**Fig. 15:**
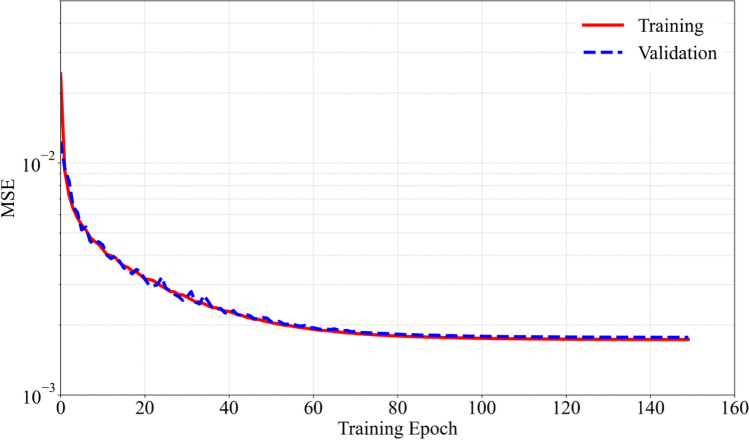
Learning curve for training and validation of the selected ANN

A schematic representation of the proposed network architecture is shown in Fig. 14. The network consists of 5 inputs, 1 output, and a total of 6 hidden layers. The first hidden layer has 2048 neurons, the 2nd layer has 1024 neurons, the 3rd and 4th layers have 512 neurons each, and the next two hidden layers have 256 and 128 neurons, respectively. The selected network is re-trained for a total of 150 epochs, and the evolution of MSE is shown in Fig. 15. It is noted that the MSE decreases gradually up until 100 epochs, after which it stays almost stable at approx. 1.7 × 10^−3^. It is also observed that both the training and validation errors are in close proximity to each other, implying a potential generalization of the ANN model.

**Fig. 16:**
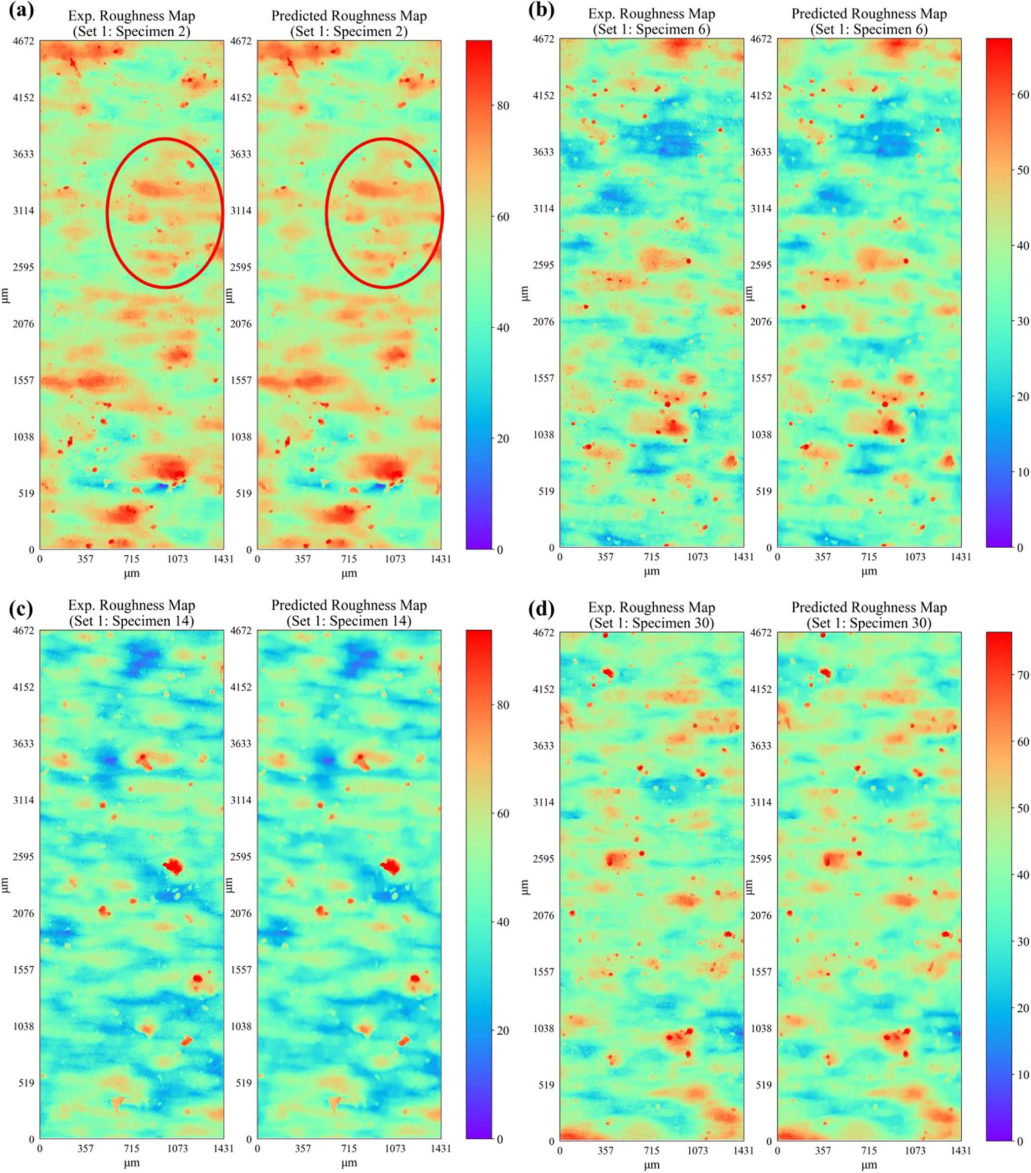
Comparison of ANN predicted and experimental surface roughness profiles for set 1 specimens

**Fig. 17:**
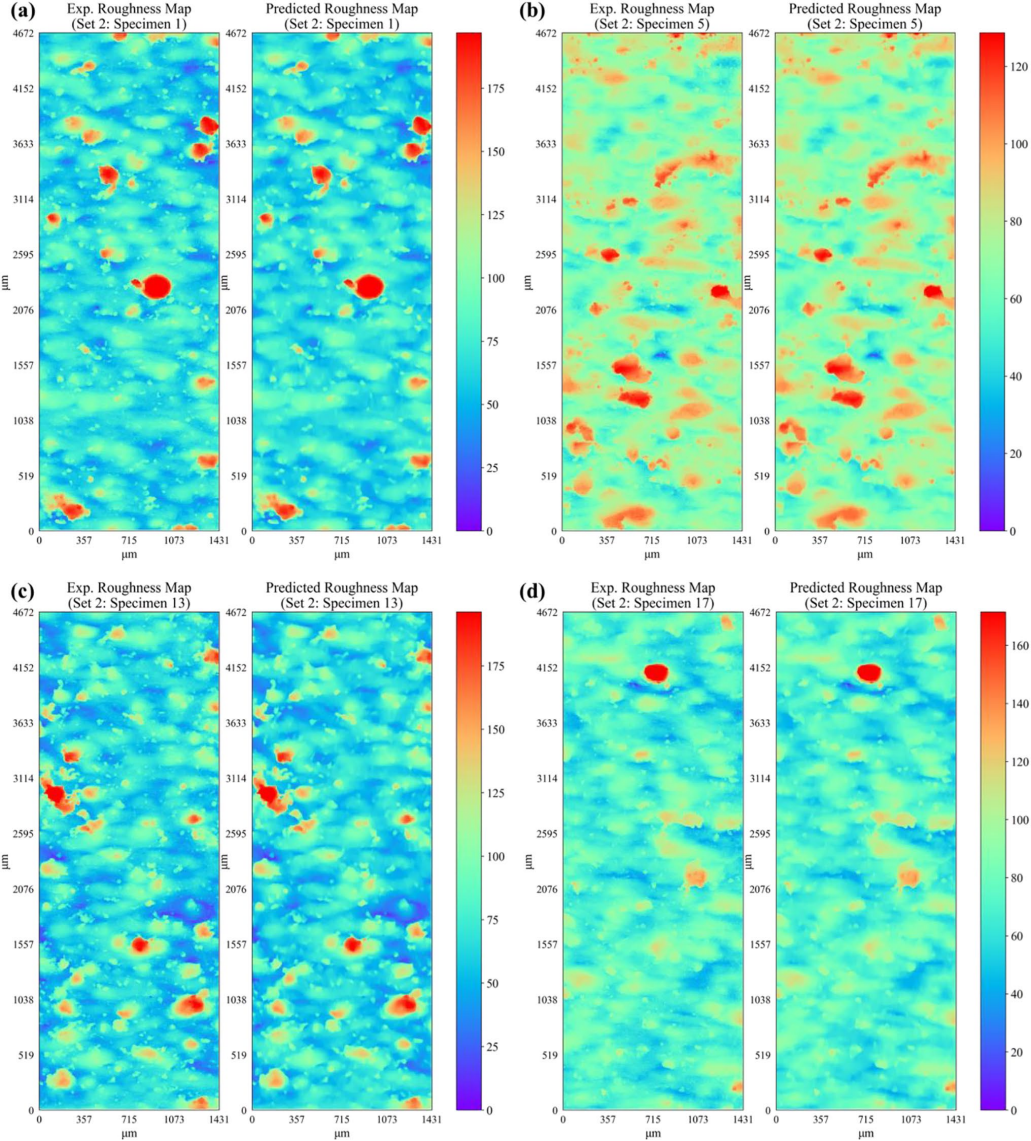
Comparison of ANN predicted and experimental surface roughness profiles for set 2 specimens

**Fig. 18:**
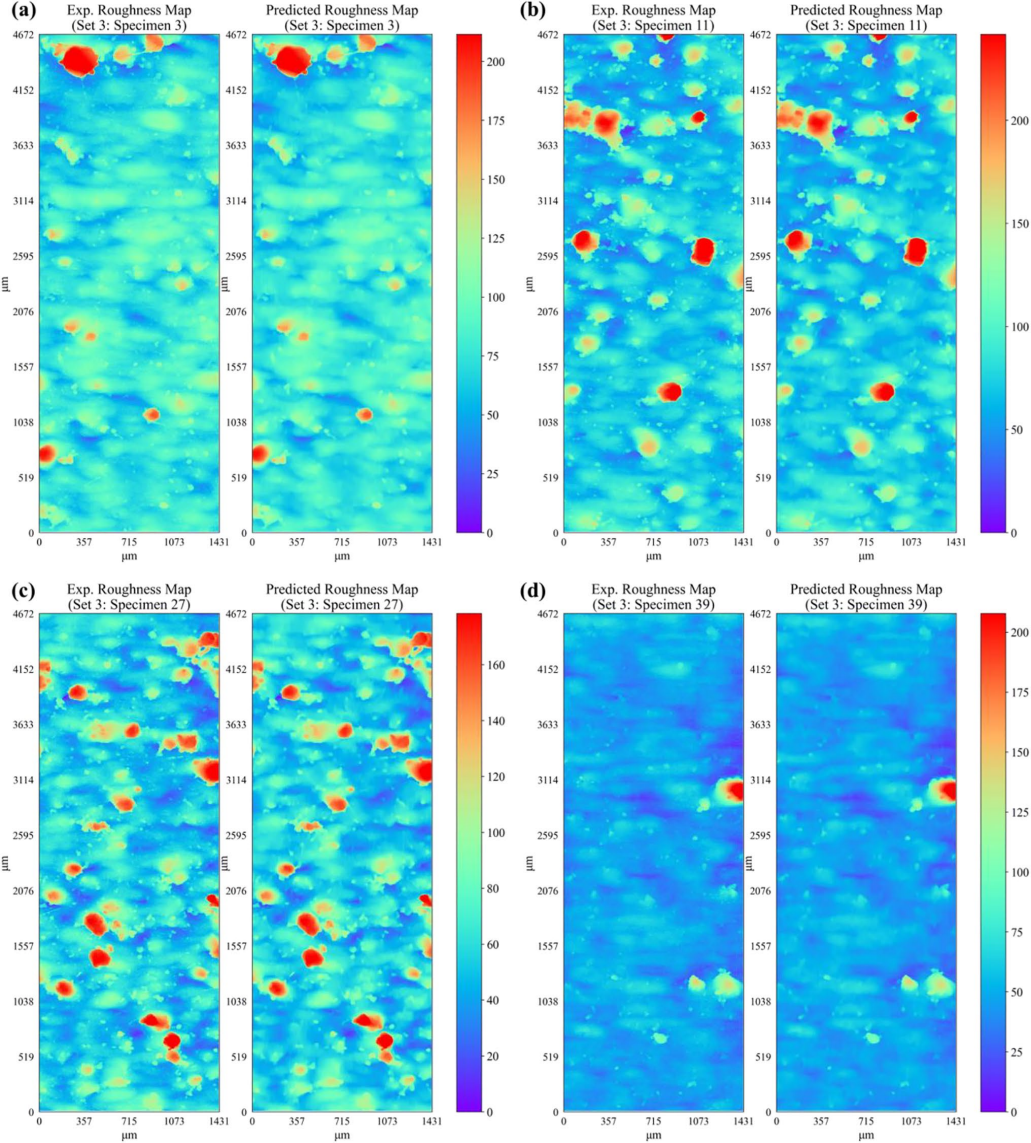
Comparison of ANN predicted and experimental surface roughness profiles for set 3 specimens

Figure 16 shows a comparison of the predicted surface roughness profile (i.e., height map $Z(x,y))$ and the corresponding experimental data for specimens in set 1. For conciseness, the profile height maps for only four representative specimens from the first three sets are presented. The corresponding root mean square error (RMSE) between the predicted and experimental roughness height map for each specimen in set 1 is given in Table 10. It is observed that the developed deep neural network shows excellent predictive capability and is able to successfully predict the overall appearance of the experimental surface map for each specimen in set 1. Apart from predicting the overall appearance of the roughness profile, the model also successfully predicts the location and intensity of the majority of the surface peaks (in dark red color) and valleys (in dark blue color). The RMSE in surface height predictions ranges between 2.5 and 3.5 μm across all specimens within set 1 (see Table 10), which is less than 4% of the maximum profile height values. Figures 17 and 18 present the comparison of the predicted surface profile with the corresponding experimental data for specimens in set 2 and set 3, respectively. The corresponding RMSE between the predicted and experimental roughness height maps for specimens within each set is given in Tables 11, 12, and 13, respectively. Consistent with the predictions of specimens within set 1, the proposed network successfully predicts the surface roughness profiles for all specimens within each set. It is important to note that the location, shape, and intensity of the surface heights are also well captured. However, some of the local variations or very fine fluctuations in height measurements are not properly predicted. For instance, upon careful observation of the encircled regions in Fig. 16a, one can see the presence of very fine local fluctuations in height measurements (i.e., characterized by fine-scale local variations in color of the contour map) that are only captured in an averaged manner by the model.

**Table 10:** Experimental and predicted surface roughness parameters for specimens in set 1

$\mathrm{Set no}.$	$\mathrm{Specimen no}.$	$\mathrm{RMSE}$ $\left(\mu m\right)$	${S}_{a} \left(\mu m\right)$ exp	${S}_{a} \left(\mu m\right)$ predict	$\mathrm{Error} \%$ ${S}_{a}$	${S}_{q} \left(\mu m\right)$ exp	${S}_{q} \left(\mu m\right)$ predict	$\mathrm{Error }\%$ ${S}_{q}$	${S}_{p} \left(\mu m\right)$ exp	${S}_{p} \left(\mu m\right)$ predict	$\mathrm{Error} \%$ ${S}_{p}$	${S}_{v} \left(\mu m\right)$ exp	${S}_{v} \left(\mu m\right)$ predict	$\mathrm{Error }\%$ ${S}_{v}$
1	2	2.44	7.17	6.90	3.79	9.19	8.85	3.64	57.35	49.96	12.90	57.20	52.80	7.69
1	6	3.09	6.65	6.16	7.44	8.49	7.91	6.90	48.86	40.18	17.75	35.36	28.37	19.76
1	10	2.83	6.71	6.30	6.19	8.65	8.15	5.70	59.47	49.67	16.48	40.17	34.38	14.42
1	14	3.40	8.37	7.93	5.25	10.75	10.22	4.98	73.85	62.50	15.37	43.29	34.62	20.00
1	22	3.53	6.37	5.70	10.46	8.21	7.40	9.90	65.40	55.23	15.54	38.83	31.42	19.06
1	26	3.18	7.01	6.48	7.53	9.46	8.89	6.03	69.75	59.26	15.04	41.95	34.05	18.81
1	30	2.88	6.49	6.03	7.11	8.44	7.94	5.94	53.68	45.01	16.14	41.79	33.72	19.29
1	38	3.01	6.57	6.09	7.27	8.45	7.89	6.60	60.13	51.58	14.21	35.29	28.20	20.08

**Table 11:** Experimental and predicted surface roughness parameters for specimens in set 2

$\mathrm{Set no}.$	$\mathrm{Specimen no}.$	$\mathrm{RMSE}$ $\left(\mu m\right)$	${S}_{a} \left(\mu m\right)$ exp	${S}_{a} \left(\mu m\right)$ predict	$\mathrm{Error} \%$ ${S}_{a}$	${S}_{q} \left(\mu m\right)$ exp	${S}_{q} \left(\mu m\right)$ predict	$\mathrm{Error }\%$ ${S}_{q}$	${S}_{p} \left(\mu m\right)$ exp	${S}_{p} \left(\mu m\right)$ predict	$\mathrm{Error} \%$ ${S}_{p}$	${S}_{v} \left(\mu m\right)$ exp	${S}_{v} \left(\mu m\right)$ predict	$\mathrm{Error }\%$ ${S}_{v}$
2	1	3.62	16.55	16.22	2.04	25.15	24.87	1.10	172.31	160.40	6.91	73.75	61.90	16.06
2	5	3.58	11.02	10.60	3.83	14.35	13.90	3.13	88.64	79.58	10.21	72.15	59.97	16.88
2	9	3.16	11.63	11.34	2.49	16.28	15.99	1.80	142.05	132.83	6.49	51.41	42.57	17.18
2	13	3.74	18.70	18.39	1.67	26.13	25.84	1.11	173.38	161.81	6.67	71.87	61.46	14.49
2	17	3.43	11.76	11.41	2.96	16.20	15.84	2.22	141.92	129.90	8.47	72.42	63.80	11.91
2	21	3.36	11.04	10.66	3.42	15.92	15.56	2.25	153.12	138.40	9.61	50.01	40.77	18.47
2	25	3.49	11.25	10.83	3.71	15.07	14.66	2.69	91.64	82.31	10.17	70.79	58.74	17.03
2	29	3.44	12.90	12.56	2.64	18.41	18.10	1.70	143.16	127.81	10.73	56.20	46.27	17.67
2	37	3.07	9.96	9.65	3.09	12.77	12.42	2.77	87.64	81.45	7.07	56.53	48.67	13.90

**Table 12:** Experimental and predicted surface roughness parameters for specimens in set 3

$\mathrm{Set no}.$	$\mathrm{Specimen no}.$	$\mathrm{RMSE}$ $\left(\mu m\right)$	${S}_{a} \left(\mu m\right)$ exp	${S}_{a} \left(\mu m\right)$ predict	$\mathrm{Error} \%$ ${S}_{a}$	${S}_{q} \left(\mu m\right)$ exp	${S}_{q} \left(\mu m\right)$ predict	$\mathrm{Error }\%$ ${S}_{q}$	${S}_{p} \left(\mu m\right)$ exp	${S}_{p} \left(\mu m\right)$ predict	$\mathrm{Error} \%$ ${S}_{p}$	${S}_{v} \left(\mu m\right)$ exp	${S}_{v} \left(\mu m\right)$ predict	$\mathrm{Error }\%$ ${S}_{v}$
3	3	3.39	15.07	14.72	2.14	23.30	23.04	1.12	184.06	169.37	7.98	80.30	69.88	12.96
3	7	3.38	9.34	8.87	5.01	13.14	12.71	3.29	117.73	108.56	7.79	53.05	46.40	12.54
3	11	3.60	21.63	21.38	1.12	33.49	33.30	0.59	222.23	217.01	2.35	79.87	69.20	13.37
3	15	3.89	9.19	8.73	4.97	12.98	12.55	3.30	112.84	101.54	10.00	55.54	48.65	12.40
3	19	3.20	8.29	7.85	5.30	10.84	1.37	4.42	95.44	88.36	7.41	47.67	41.38	13.19
3	23	3.42	9.70	9.22	5.01	15.47	15.08	2.55	185.79	177.36	4.54	56.52	50.79	10.14
3	27	3.46	19.36	19.10	1.35	28.04	27.82	0.79	159.53	145.13	9.03	63.35	57.36	9.46
3	31	3.29	9.96	9.57	3.90	14.13	13.75	2.65	113.13	1.1.27	10.47	56.52	48.40	14.36
3	39	3.18	8.38	7.92	5.55	14.73	14.41	2.17	211.24	196.22	7.11	48.46	40.98	15.42

**Table 13:** Experimental and predicted surface roughness parameters for specimens in set 4

$\mathrm{Set no}.$	$\mathrm{Specimen no}.$	$\mathrm{RMSE}$ $\left(\mu m\right)$	${S}_{a} \left(\mu m\right)$ exp	${S}_{a} \left(\mu m\right)$ predict	$\mathrm{Error} \%$ ${S}_{a}$	${S}_{q} \left(\mu m\right)$ exp	${S}_{q} \left(\mu m\right)$ predict	$\mathrm{Error }\%$ ${S}_{q}$	${S}_{p} \left(\mu m\right)$ exp	${S}_{p} \left(\mu m\right)$ predict	$\mathrm{Error} \%$ ${S}_{p}$	${S}_{v} \left(\mu m\right)$ exp	${S}_{v} \left(\mu m\right)$ predict	$\mathrm{Error }\%$ ${S}_{v}$
4	4	3.37	12.06	11.70	2.95	17.76	17.44	1.80	133.68	120.12	10.15	54.53	45.47	16.60
4	8	3.44	11.55	11.18	3.23	16.96	16.63	1.94	157.48	143.07	9.15	69.50	56.00	19.42
4	12	3.99	14.56	14.14	2.88	21.29	20.93	1.70	158.29	145.39	8.15	66.67	54.14	18.79
4	16	4.88	14.42	13.73	4.78	20.73	20.13	2.89	184.96	167.17	9.62	63.73	52.73	17.25
4	20	3.29	9.58	9.16	4.39	12.97	12.56	3.12	85.33	77.52	9.15	50.18	42.01	16.27
4	24	3.50	10.98	10.57	3.74	15.52	15.11	2.62	152.53	137.82	9.64	47.44	39.68	16.35
4	28	3.56	20.21	19.98	1.17	29.70	29.50	0.67	176.08	158.62	9.91	64.88	56.08	13.56
4	32	3.63	18.30	18.02	1.53	26.63	26.39	0.90	172.22	155.55	9.68	65.34	57.02	12.73
4	40	3.61	10.92	10.48	3.99	15.84	15.42	2.64	161.98	148.23	8.48	54.55	44.25	18.87

As mentioned previously, the predicted roughness profile height $Z(x,y)$ results are further processed using Eqs. (1) to (4) to compute the well-known surface roughness parameters. The predicted surface roughness parameters along with the corresponding experimental data are given in Tables 10, 11,  12, and 13 for specimens in set 1, set 2, set 3, and set 4, respectively. It is observed that the predicted surface roughness parameters across all four printed sets show good conformity to the corresponding experimental values. The prediction error in ${S}_{a}$ and ${S}_{q}$ is less than 10% for all specimens in set 1 and less than 4% for majority of the specimens in set 2, set 3, and set 4. This highlights the capability of the network to successfully predict the important surface roughness parameters such as ${S}_{a}$ and ${S}_{q}$. The prediction error for the maximum peak height ${S}_{p}$ and the maximum valley depth ${S}_{v}$ are within the range of 5 to 20% in most cases. Since ${S}_{p}$ and ${S}_{v}$ are derived based on a single value (i.e., maximum peak or valley) from the roughness height map, the error in predictions of these parameters is always greater than that for ${S}_{a}$ and ${S}_{q}$. Nevertheless, the ANN model is successful in predicting the typical roughness-related parameters with reasonable accuracy. It is also noted that the prediction error in surface roughness parameters is higher for specimens in set 1 as compared to the other three sets. This is possibly due to the fact that specimens in set 1 have significantly lower roughness as compared to the specimens in the other three sets. Hence, during training, the learning behavior of the network tends to be slightly more biased toward the later three sets due to the combined high number of specimens compared to set 1. It is also worth mentioning that the ANN model is also able to successfully predict the surface roughness of specimens that are located at the right or leftmost edges of the build plate (i.e., specimen 14 from set 1; specimen 1 and specimen 13 from set 2; specimen 3, specimen 11, and specimen 27 from set 3; specimen 12, specimen 16, specimen 28, and specimen 32 from set 4) and have higher roughness compared to the rest of the specimens within their corresponding sets. Thus, the proposed network is able to capture the effects of specimen location on the resulting surface roughness.

**Fig. 19:**
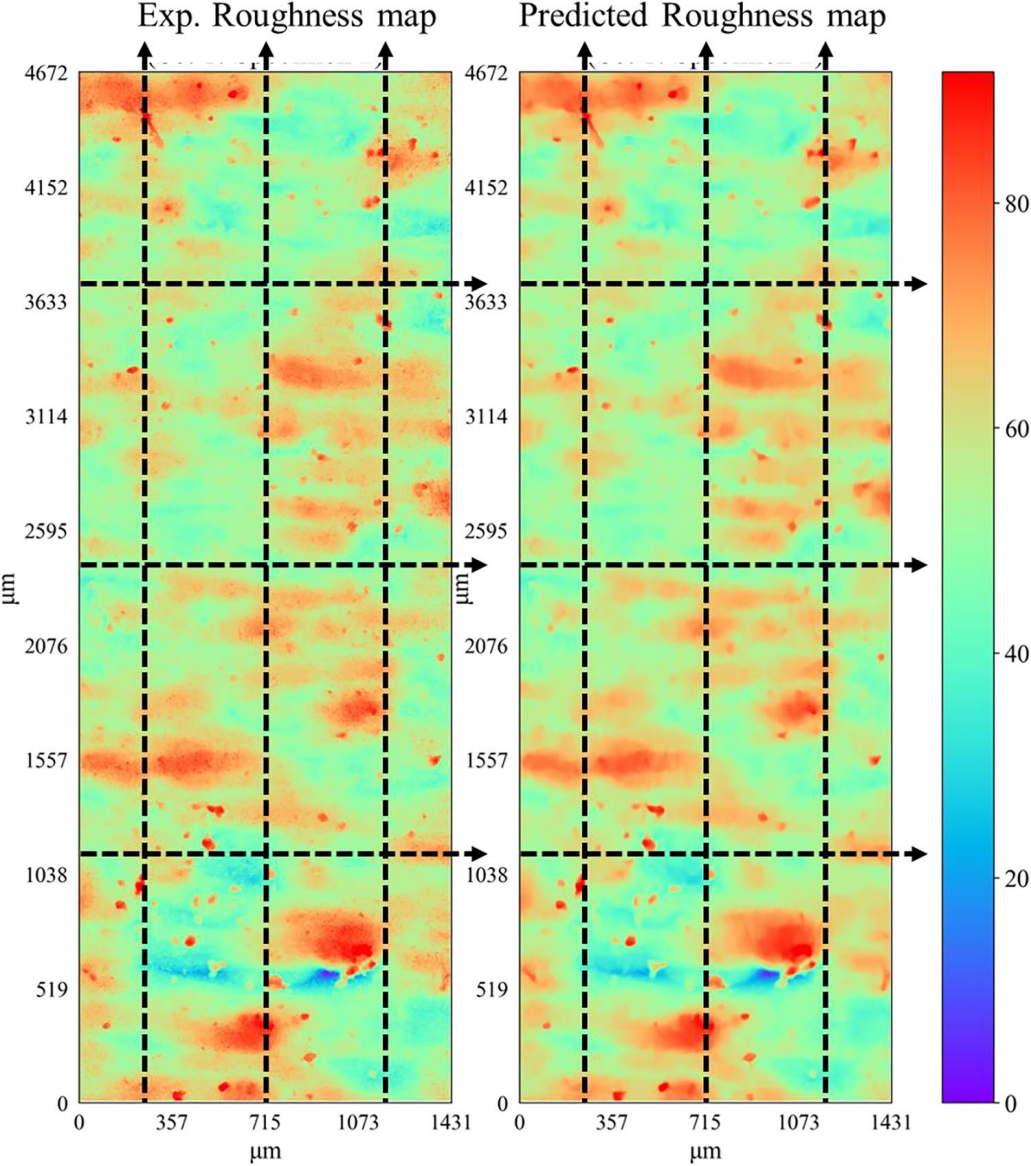
Schematic representation of 3 horizontal and 3 vertical roughness line scans

**Fig. 20:**
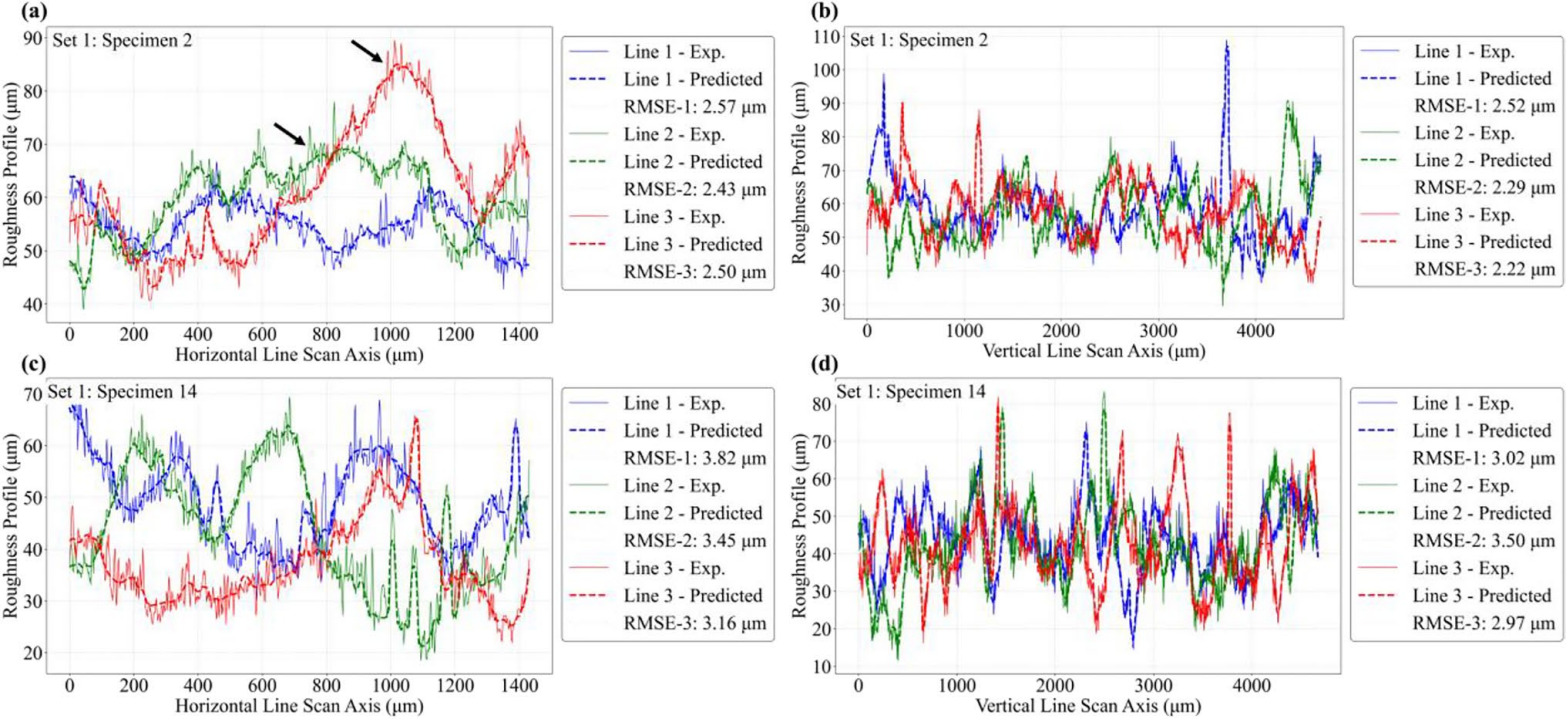
Comparison of local roughness predictions with experimental data using line scans for set 1 specimens

**Fig. 21:**
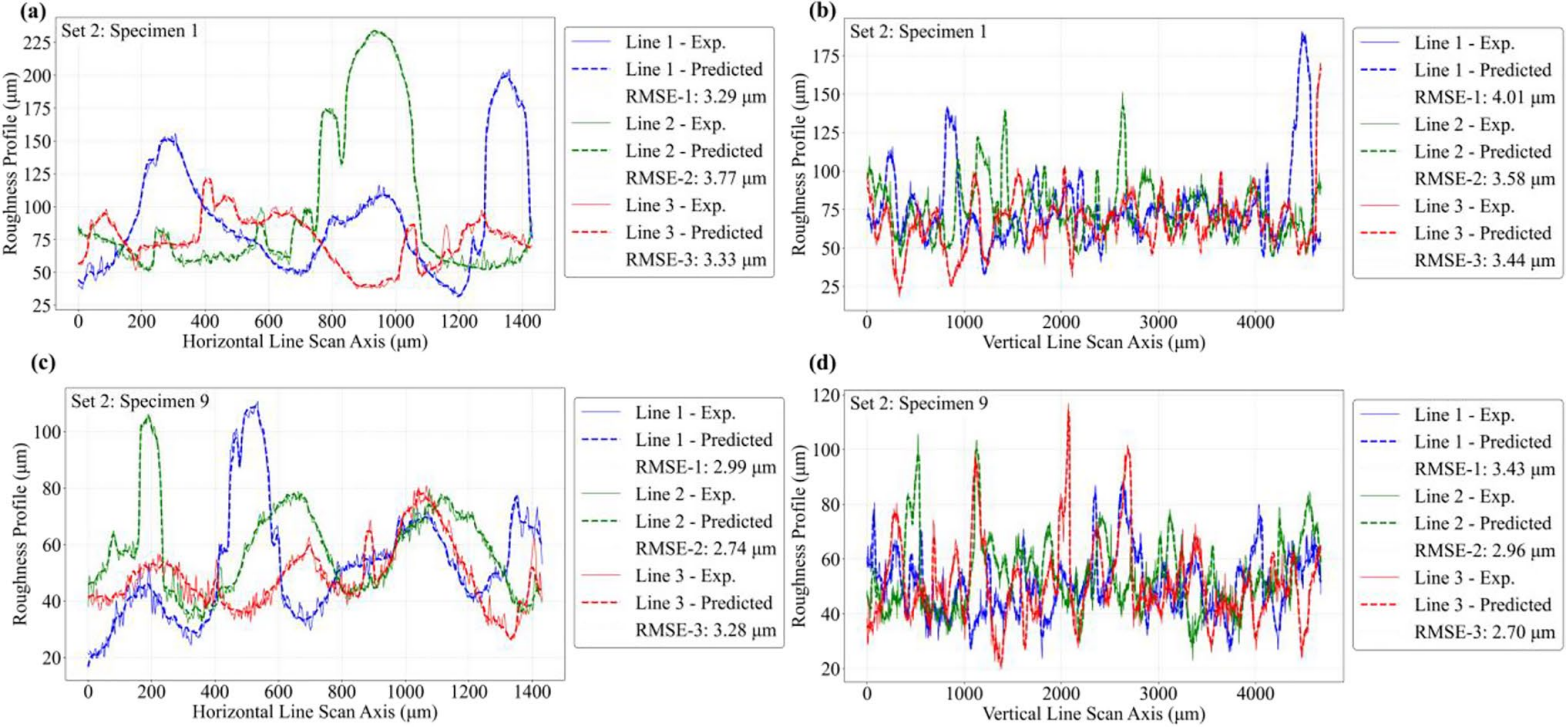
Comparison of local roughness predictions with experimental data using line scans for set 2 specimens

**Fig. 22:**
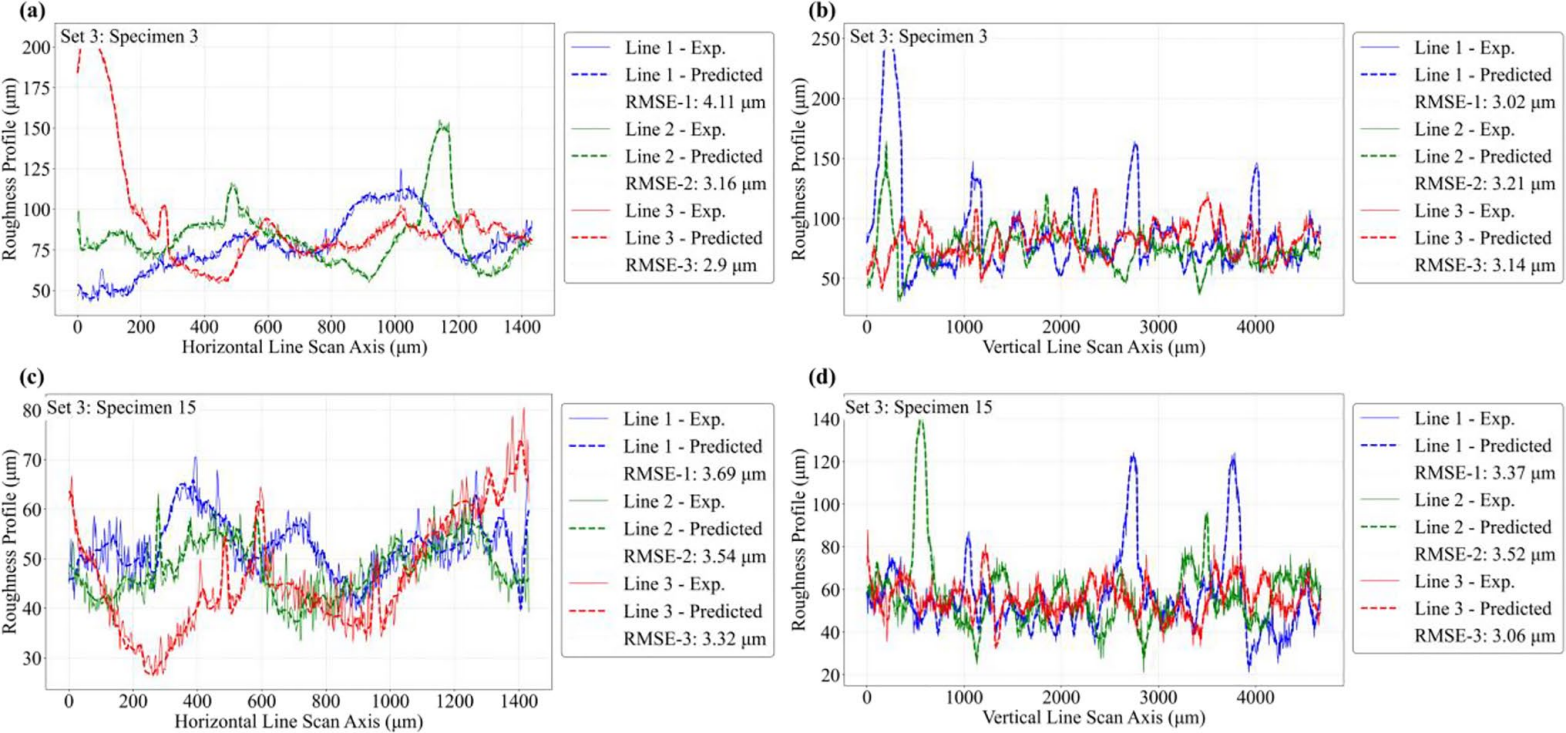
Comparison of local roughness predictions with experimental data using line scans for set 3 specimens

The predictive capability of the ANN model is further investigated by comparing roughness line scan results against the corresponding experimental data. For this purpose, the roughness height profiles are extracted along several line scans across the length and width of the roughness profile maps, as shown schematically in Fig. 19. Three horizontal and three vertical line scans are extracted using the same *x*–*y* coordinate locations across all specimens, and the results are compared against experimental data. Figures 20, 21, and 22 compare the predicted and experimental roughness line scan measurements for specimens in set 1, set 2, and set 3, respectively. For simplicity, comparison results are only shown for four specimens for the first three printed sets. The RMSE for each line scan is also reported within the legend of each figure. It is observed that the predicted line scan topography results are in excellent agreement with the corresponding experimental data across all specimens. This is further confirmed by the rather low RMSE values ranging between 2.5 and 3.5 μm for all specimens. Furthermore, it is noted that the model can successfully capture all major fluctuations in surface topography and is also able to properly capture the widths of such fluctuations. However, there are some very fine-scale fluctuations (i.e., see arrows in Fig. 20a) in the surface height measurements that the ANN is not able to capture properly. Nevertheless, the proposed model can successfully predict the key aspects of topographical behavior.

In the present work, a numerical approach is developed for the prediction of surface topography and typical surface roughness parameters using a deep learning framework. The ability of the presented framework to effectively predict local variations in surface topography is very promising. It is noted that although powder layer thickness was the same among different sets of specimens, it was included as a feature set in the present study for easier generalization of the network for further development. Since AM printing process involves several key parameters and the process-induced surface roughness is controlled by the synergistic effects of such parameters, the present model may be extended to include the effects of such parameters in the model as the experimental data becomes available. This would likely improve the predictions and further promote the general applicability of the model. The current framework with further extensions may be applied to predict surface roughness along the inner wall of hollow channels that is almost impossible to be measured experimentally using non-destructive means. Lastly, the successful application of the present approach will further promote the application of such methods toward material development and process optimization in AM.

## Summary and conclusions

In the current work, a deep learning framework is developed to predict the process-induced surface roughness of AlSi10Mg aluminum alloy fabricated using LPBF. The framework involves the fabrication of round bar AlSi10Mg specimens using LPBF, surface roughness measurement using 3D laser scanning profilometry, extraction, coupling, and streamlining of roughness and LPBF processing data, feature engineering to choose the most compact and relevant features and the development, validation, and evaluation of an ANN-based deep learning model. A mix of core and contour-border laser scanning strategies are employed with varying laser power and scanning speeds to fabricate four sets of specimens with different surface roughness conditions. The experimental roughness data combined with laser processing parameters are used to train and validate a deep learning neural network. The proposed network consists of 5 inputs, 1 output, and a total of 6 hidden layers. The inputs to the network are the contour-border laser scanning and AM process-related parameters. The network employs a hyperbolic tangent as an activation function and uses the Adam adaptive learning algorithm for optimization and is trained for a total of 150 epochs. The developed framework predicts the surface topography and is employed to predict surface roughness parameters. Some of the key observations and conclusions are as follows.

A mix of core and border-contour rescanning strategies can be successfully employed to achieve lower surface roughness for AlSi10Mg aluminum specimens fabricated using LPBF. Surface roughness as low as ${S}_{a}\approx 6.5\mathrm{ \mu m}$ is successfully achieved in the present work by varying the laser power and scanning speeds.

The choice of core scanning parameters has no significant impact on surface roughness. The surface roughness is mainly controlled by the contour-border scanning parameters.

The use of higher linear energy density (LED) leads to lower surface roughness. However, the actual surface roughness is a consequence of the synergistic effects of several different process parameters and may not be properly represented by simple factors such as the LED alone.

Apart from the LPBF process parameters, the surface roughness is also affected the specimen location on the build plate. In the present work, the specimens in the vicinity of the right or leftmost edges of the build plate have comparatively higher roughness compared to other specimens within the same print set. This behavior is most likely associated with the directional flow of the inert gas within the build chamber and the spattering of powder particles. The spattering and accumulation of powder particles could lead to powder agglomeration, loss of powder in some areas, and redistribution of the powder on the powder bed, which can alter the processing of subsequent laser tracks and powder layers, thereby impacting the surface roughness.

The proposed deep learning framework successfully predicts the surface roughness profiles for the four sets of specimens fabricated using different scanning parameters. The intensity and location of the surface peaks and valleys as well as their shapes are well predicted. The predicted surface roughness ($${S}_{a})$$ measurements are well within 5% of experimental error for the majority of the specimens. The ability of the proposed framework to successfully predict local variations in surface topography is very promising, as demonstrated by comparing roughness line scan results with corresponding experimental data.

An important outcome of the present work is the proof of feasibility that a machine learning-based deep neural network can be built in such a way that predicts several important aspects of surface topography induced by LPBF-based additive manufacturing. The research presented herein motivates further applications of such machine learning-based methods toward material development and AM process optimization.

## Data Availability

The raw/processed data required to reproduce these findings cannot be shared at this time as the data also forms part of an ongoing study.
